# Functional Disruption of the Cancer‐Relevant Interaction between Survivin and Histone H3 with a Guanidiniocarbonyl Pyrrole Ligand

**DOI:** 10.1002/anie.201915400

**Published:** 2020-01-28

**Authors:** Cecilia Vallet, Dennis Aschmann, Christine Beuck, Matthias Killa, Annika Meiners, Marcel Mertel, Martin Ehlers, Peter Bayer, Carsten Schmuck, Michael Giese, Shirley K. Knauer

**Affiliations:** ^1^ Department of Molecular Biology II University of Duisburg-Essen Universitätsstraße 5 45141 Essen Germany; ^2^ Institute for Organic Chemistry University of Duisburg-Essen Germany; ^3^ Department of Structural and Medicinal Biochemistry University of Duisburg-Essen Germany

**Keywords:** cancer, molecular recognition, PPI inhibitors, supramolecular chemistry, survivin

## Abstract

The protein Survivin is highly upregulated in most cancers and considered to be a key player in carcinogenesis. We explored a supramolecular approach to address Survivin as a drug target by inhibiting the protein–protein interaction of Survivin and its functionally relevant binding partner Histone H3. Ligand **L1** is based on the guanidiniocarbonyl pyrrole cation and serves as a highly specific anion binder in order to target the interaction between Survivin and Histone H3. NMR titration confirmed binding of **L1** to Survivin's Histone H3 binding site. The inhibition of the Survivin–Histone H3 interaction and consequently a reduction of cancer cell proliferation were demonstrated by microscopic and cellular assays.

## Graphical abstract

**Effective interruption**: The supramolecular guanidiniocarbonyl pyrrole ligand L1 targets the Histone H3 binding site of the protein Survivin, which is highly upregulated in cancer. Inhibiting the interaction between the two proteins successfully reduced cancer cell proliferation.

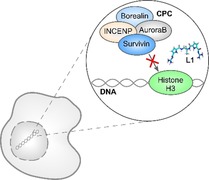

Survivin is overexpressed in almost all malignant tumors and is considered an early diagnostic and prognostic biomarker.^1^ The protein has been associated with a resistance against chemo‐ and radiotherapy and a poor clinical outcome.^2^ Survivin is involved in two key processes of carcinogenesis: As a member of the inhibitor of apoptosis protein (IAP) family, it counteracts cell death, and as part of the chromosomal passenger complex (CPC) it promotes cell proliferation.^3^ As Survivin is mainly expressed during embryonic development but mostly absent in terminally differentiated adult tissues, it is considered to be one of the most cancer‐specific proteins identified so far.^4^ However, Survivin possesses no enzymatic activity, which makes it challenging to address the protein as a drug target. Current therapeutic strategies include antisense oligonucleotides, siRNAs, small‐molecule inhibitors, gene therapy, and immunotherapy but none of these approaches has yet reached the clinic.^5^ We aimed to identify a ligand that specifically interferes with the protein–protein interaction (PPI) between Survivin and its functionally relevant binding partner Histone H3 (Figure 1). Twenty years ago, PPIs were still thought to be “intractable” as PPI interfaces are, in contrast to the deep cavities that typically bind small molecules, flat and large.^6^ Recently, the modulation of PPIs has shown more and more promising results since supramolecular chemistry has emerged as a novel tool to target protein interfaces.^7^, ^8^ However, the design of ligands, which specifically address a well‐defined hot spot on the protein surface, remains challenging.^9^

**Figure 1:**
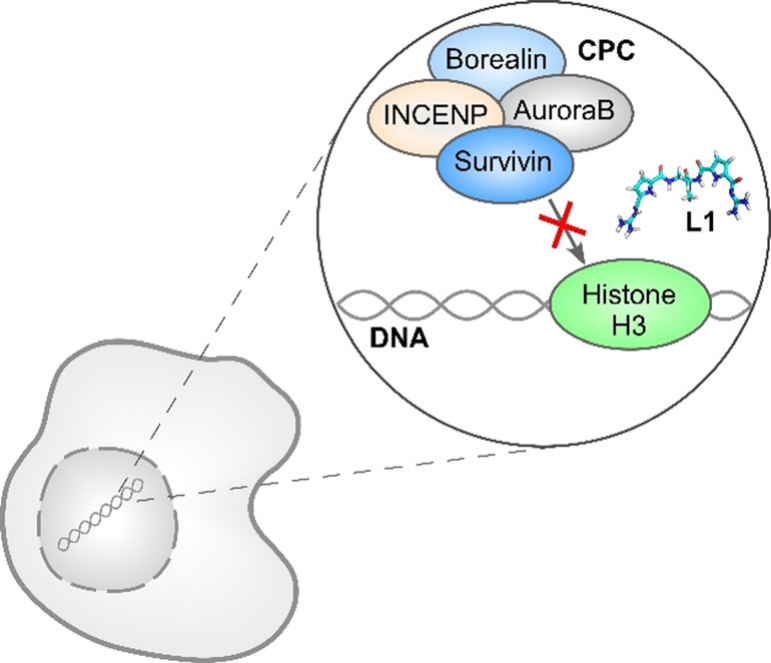
The interaction between Survivin (blue) and Histone H3 (green) is essential for Survivin to fulfill its role in mitosis as a member of the chromosomal passenger complex (CPC), which consists of Survivin, Borealin (light blue), AuroraB (gray), and INCENP (yellow). The supramolecular guanidiniocarbonyl pyrrole cation (GCP) ligand **L1** (turquoise) was designed to inhibit the interaction between Survivin and Histone H3 and thereby decrease cancer cell proliferation.

The most successful approaches so far have used compounds that display specific, well‐characterized recognition properties for amino acids and peptides in order to functionally modulate PPIs.^7^ Some well‐known examples are calixerenes that were linked to peptide loops to mimic the structure of antibodies and inhibit the interaction between cytochrome c and the cytochrome c peroxidase, cucurbiturils that have been used to induce and reversibly control the dimerization of proteins, and lysine‐ and arginine‐specific molecular tweezers that are able to disrupt the PPI between 14‐3‐3 and its partner proteins C‐Raf and ExoS.^10^ We show a proof‐of‐concept using supramolecular chemistry to design a ligand based on the guanidiniocarbonyl pyrrole cation (GCP) in order to target the interaction between Survivin and Histone H3. The guanidinium moiety in the form of arginine is not only present in the active sites of many enzymes as a binder for anionic substrates but has in addition proven to be an excellent binding motif in supramolecular chemistry. Guanidinium scaffolds have been used to develop artificial receptors that are able to bind oxoanions through hydrogen bonding as well as hydrophobic and charge‐pairing interactions.^11^ The cationic guanidiniocarbonyl pyrrole (GCP) is a rigid planar analogue with superior binding properties in aqueous solvents containing competing anions and salts, which makes it suitable for applications in a cellular environment. We have already used it for the design of artificial receptors for amino acids, oligopeptides, and oligonucleotides and in artificial transfection vectors for gene delivery.^12^

To identify ligands suitable to target the surface‐exposed anionic Histone H3 binding site of Survivin, we performed docking studies with a focused library of ligands containing one or two GCP units to address the negatively charged amino acids on the protein surface. We tested different linkers to vary the distance between the GCP units in order to achieve the best binding orientation between ligands and target amino acids and identified ligand 1 (**L1**) as the ligand with the best docking score (Figure 2 A,B).

**Figure 2:**
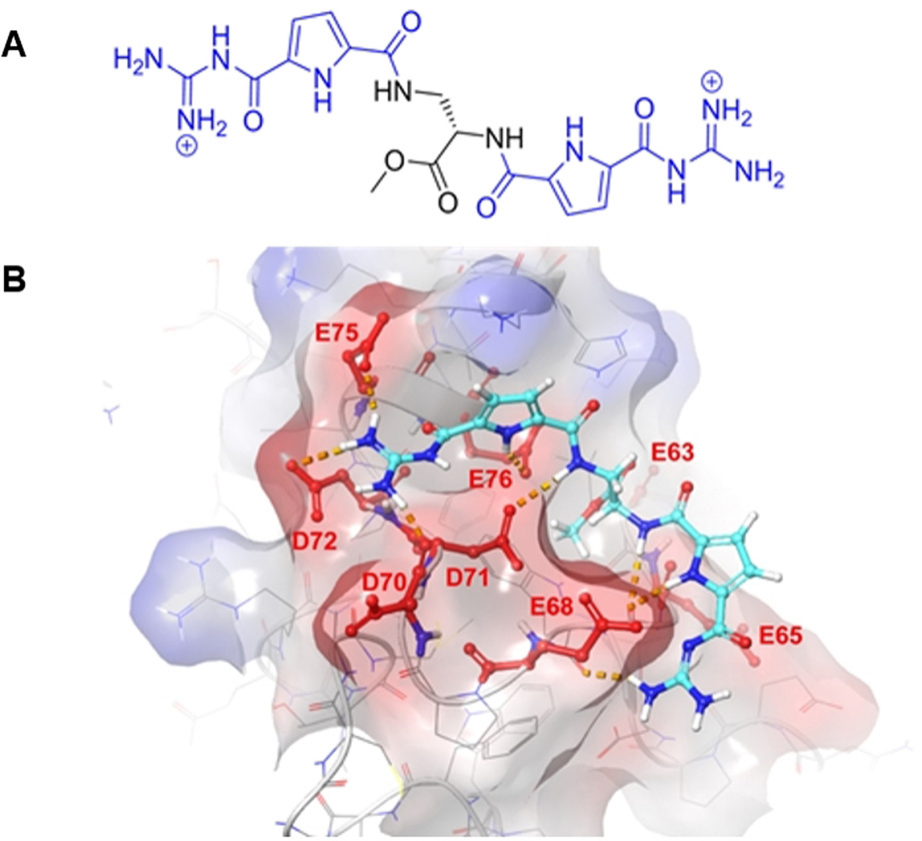
A) Chemical structure of the supramolecular GCP ligand 1 (**L1**) with two guanidiniocarbonyl pyrrole (GCP) groups (blue). B) Docking of **L1** (turquoise) to Survivin's Histone H3 binding site. The dotted lines (orange) indicate interactions between ligand and protein interaction sites of the ligand are highlighted in red.

To map the binding of **L1** to distinct residues on the protein surface, we performed NMR titration experiments. The titration resulted in chemical shift perturbations at glutamic acids (E) 65 and 68, aspartic acids (D) 70, 71, and 72, and glutamic acids (E) 75 and 76 (Figure 3 A). Those amino acids correspond to the known Histone H3 binding site of Survivin, which comprises amino acids 51 to 80.^13^ Furthermore, a decrease in NMR signal intensities indicates a binding equilibrium with kinetics in the intermediate time regime (ms), which corresponds to a dissociation constant in the μm range. Indeed, also the relative intensities *I*/*I*_0_ showed a significant decrease within Survivin's Histone H3 binding site, thereby indicating ligand binding within this region. The intensities strongly decreased for glutamic acid (E) 68, aspartic acid (D) 70, 71, and 72, and glutamic acid (E) 75 (Figure 3 B).

**Figure 3:**
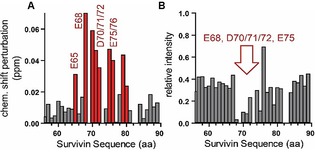
NMR titration experiments confirmed binding of **L1** to Survivin's Histone H3 binding site. A) Chemical shift perturbation (CSP, Δδ) of ^15^N‐labeled Survivin 1–120 (300 μm) with **L1** (300 μm), compared to protein without **L1**, plotted against the Survivin sequence. **L1**‐binding residues with prominent shift perturbations are colored in red, featuring aspartic and glutamic acid residues within the Histone H3 binding site of Survivin. B) The relative signal intensities show a significant decrease within Survivin's Histone H3 binding site indicating ligand binding within this region (red arrow). The histograms in (A) and (B) only show the region around the H3 binding site; for the full plots see Figure S1.

These results clearly show that **L1** interacts with Survivin's Histone H3 binding site. Whether **L1** is taken up by cells and able to inhibit the interaction between the two proteins in a cellular environment was verified with two different approaches. First, we performed a co‐immunoprecipitation. For this assay, HeLa cells, which are derived from cervical cancer, were transfected with HA‐tagged Survivin and treated with different ligand concentrations or the respective amount of DMSO as a control. After 24 h of incubation, cell lysates were generated and incubated with magnetic HA‐antibody‐coupled beads, which allowed the elution of Survivin–HA together with all other proteins bound to Survivin including Histone H3. The amount of Histone H3 bound to Survivin was then quantified via western blot analysis. We were able to show that **L1** indeed reduced the interaction between Survivin and Histone H3 in a concentration‐dependent manner. A ligand concentration of 10 μm already led to a 50 % decrease in Survivin–Histone H3 interaction, while a concentration of 50 μm caused a decrease of 65 % (Figure 4 A,B).

**Figure 4:**
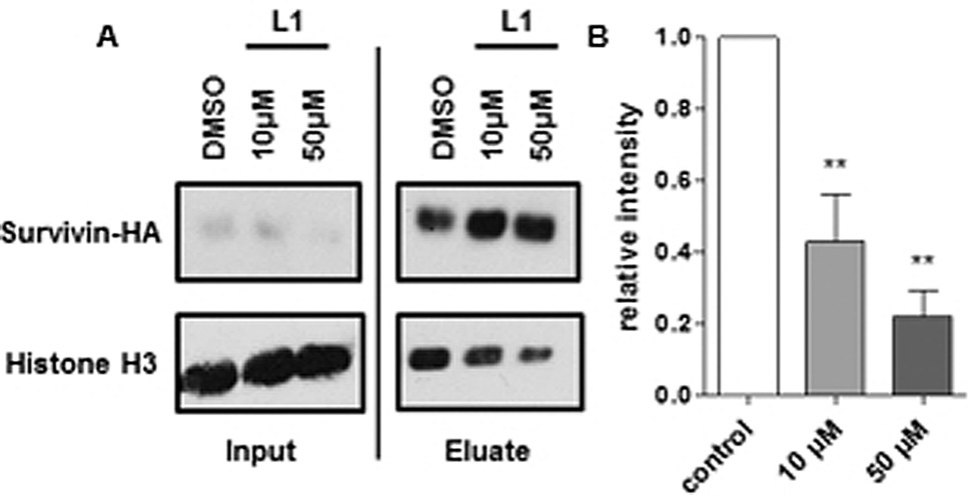
**L1** is able to inhibit the interaction between Survivin and Histone H3 in HeLa cells. A) Immunoprecipitation experiments reveal a concentration‐dependent inhibition of the Survivin–Histone H3 interaction by **L1**. B) Quantitative analysis of the western blot shows the intensity of the Histone H3 signal in the eluate normalized to the respective Survivin–HA signal of HeLa cells treated with either 10 μm **L1**, 50 μm **L1** or DMSO (control). Experiments were performed in triplicate. The error bars show the standard error of the mean. Data was analyzed by t test. Two asterisks (**) indicate a *p* value smaller than 0.01.

Since western blot analysis is considered to be only semiquantitative and co‐immunoprecipitation was performed with overexpressed and HA‐tagged Survivin, which does not fully correspond to the natural conditions inside the cell, we verified the results with an in situ proximity ligation assay (PLA). The PLA allows the visualization of protein–protein interactions within cells on an endogenous level. At first, two primary antibodies bind to the target proteins Survivin and Histone H3. Secondary antibodies that are conjugated to a matched pair of short single‐stranded oligonucleotides then recognize these antibodies. If the two targets are in close proximity (<40 nm), the oligonucleotides hybridize and ligate with two additional connector oligonucleotides to form a continuous circular DNA structure. DNA polymerase then amplifies these circular structures through rolling‐circle amplification with fluorescent nucleotides that can be detected as PLA signals with fluorescence microscopy. The PLA indeed revealed a significant decrease of the Survivin–Histone H3 interaction inside the cell upon **L1** treatment and thereby confirmed the results of the co‐immunoprecipitation (Figure 5 A,B).

**Figure 5:**
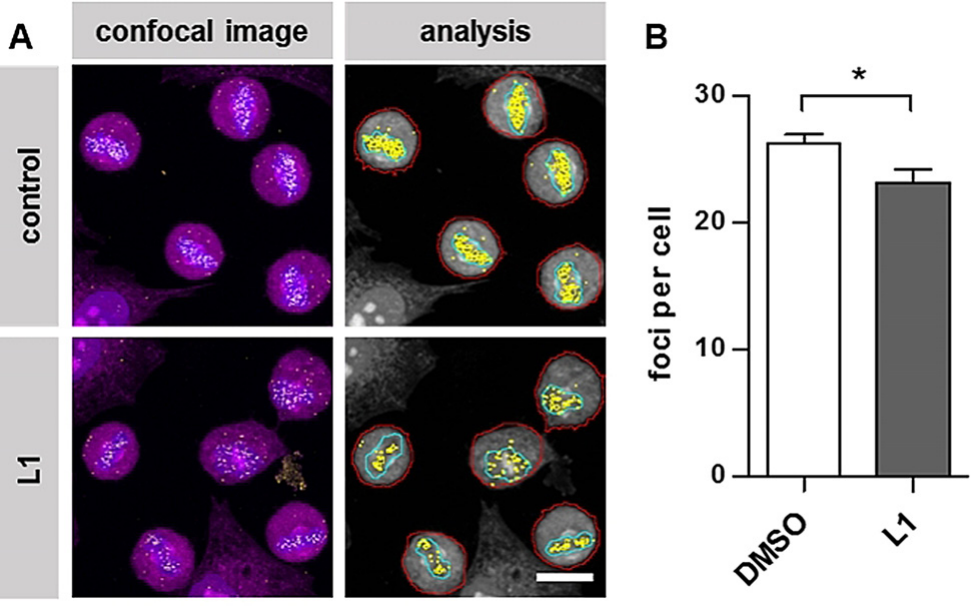
The inhibiting effect of **L1** could be confirmed via PLA. A) PLA performed in Hela cells treated with 50 μm **L1** or the respective amount of DMSO (control). Entire cells are depicted in magenta, DNA in blue, and PLA foci in yellow. Scale bar: 20 μm. B) Quantification of the PLA. The error bars show the standard error of the mean. *N*>55. Data was analyzed by t test. One asterisk (*) indicates a *p* value smaller than 0.05.

As **L1** could be shown to inhibit the interaction between Survivin and Histone H3 inside the cell, we wanted to investigate whether the inhibitor would consequently also interfere with Survivin's role in cell proliferation. Therefore, HeLa cells were treated with 50 μm of **L1** and synchronized before they were fixed during mitosis. The cells were then immunostained to allow the microscopic identification of mitotic defects. Cells that displayed mitotic defects were assigned to one of the five following categories: Multipolar Pro‐/Metaphase, Multipolar Ana‐/Telophase, Lagging Chromosomes, Acentric Fragments, and Chromatin Bridges (Figure S23).^14^ The experiments revealed that ligand treatment drastically increased the number of mitotic defects in HeLa cells. While only 6 % of control cells had mitotic defects, the amount increased to 32 % in ligand‐treated cells (Figure 6 A). Furthermore, **L1**‐treated cells showed a larger variety of mitotic defects in comparison to the control. In addition to acentric fragments and lagging chromosomes, also chromatin bridges and multipolar spindles could be observed (Figure 6 B). This confirms that **L1** is able to interfere with Survivin's mitotic functions. We quantified the inhibiting effect of the ligand on cell proliferation by performing cell proliferation assays in different types of cancer cells: HeLa cells that are derived from cervical cancer, A549 cells as a model for lung cancer, MDA‐MB‐231 cells originating from breast cancer, and HCT 116 cells that serve as a model for colon cancer.

**Figure 6:**
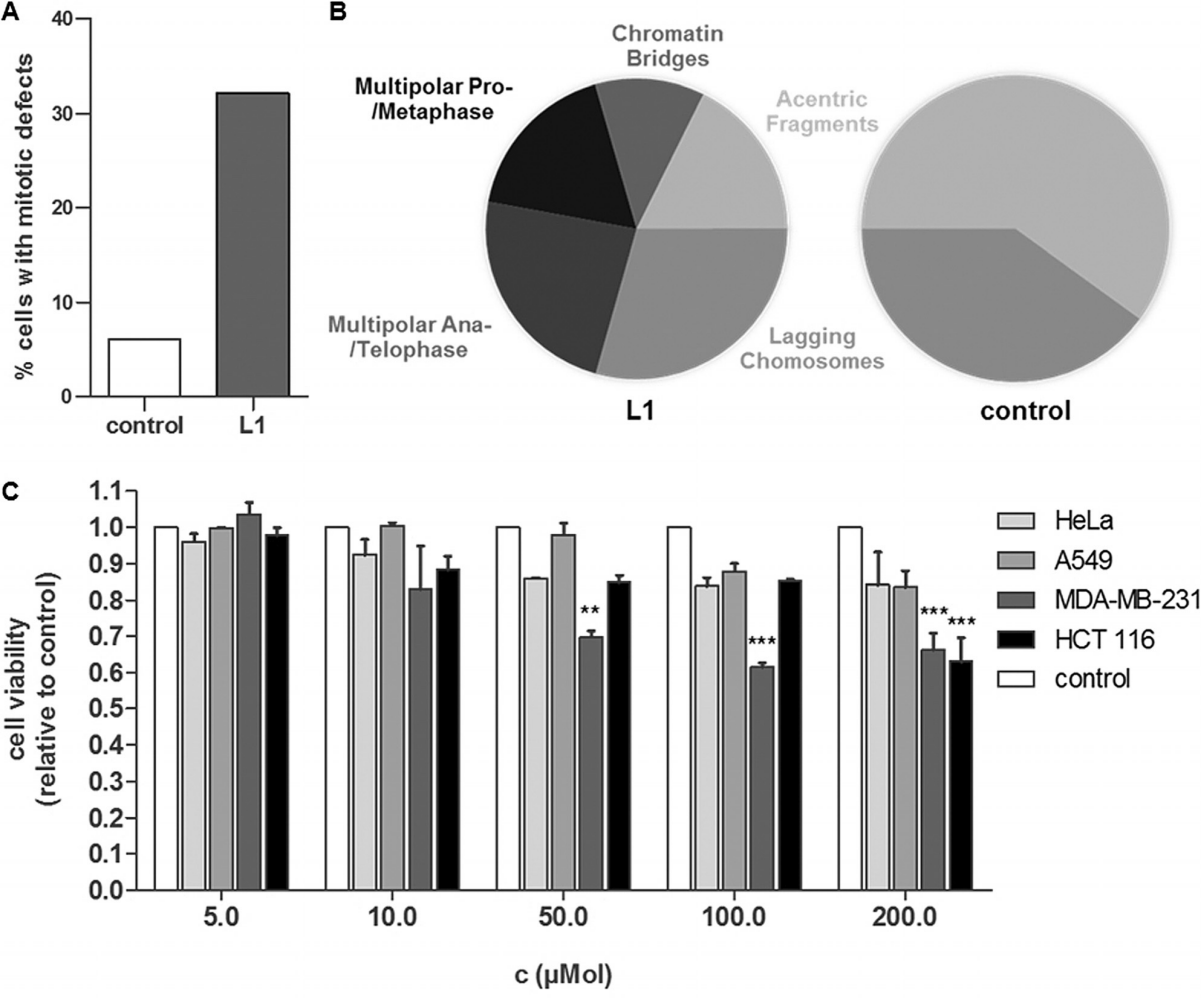
**L1** induces mitotic defects in HeLa cells and inhibits cell proliferation. A) Percentage of HeLa cells with mitotic defects in cells treated with 50 μM **L1** for 48 h in comparison to the DMSO control. B) Pie charts show proportions of the different mitotic defects found in **L1**‐treated cells in comparison to DMSO‐treated cells (control). *N*>100. C) HeLa, A549, MDA‐MB‐231, and HCT 116 cells were treated with different ligand concentrations or the respective amounts of DMSO and incubated for 72 h before cell proliferation was measured via an MTS assay. The measured absorbance at 490 nm in **L1**‐treated cells was normalized to the absorbance in DMSO‐treated cells of the respective cell type (control). Experiments were performed in triplicate. The error bars show the standard error of the mean. Data was analyzed by a 1way ANOVA test followed by a Tukey's Multiple Comparison test. Two asterisks (**) indicate a *p* value smaller than 0.01. Three asterisks (***) indicate a *p* value smaller than 0.001.

The tetrazolium compound [3‐(4,5‐dimethylthiazol‐2‐yl)‐5‐(3‐carboxymethoxyphenyl)‐2‐(4‐sulfophenyl)‐2*H*‐tetrazolium (MTS) used for this assay is added to the cells and bioreduced into a colored formazan product. The quantity of formazan product measured at 490 nm absorbance is directly proportional to the number of living cells in culture. The assay revealed that **L1** was able to successfully inhibit cell proliferation in all cancer cell lines tested (Figure 6 C). In three of the four cell lines, proliferation could be decreased by more than 30 %, which is comparable to the decrease in proliferation observed in Survivin‐depleted cells.^15^ The inhibition of cell proliferation occurred in a concentration‐dependent manner and the largest effects could be observed in the breast cancer cell line MDA‐MB‐231 and the colon cancer cell line HCT 116.

To confirm that the observed effects of **L1** on cancer cell proliferation are Survivin‐specific, we performed a rescue experiment in which we transiently transfected HCT 116 cells with Survivin–HA (Rescue) or with GFP (Control). After treating the cells with **L1** for 72 h we measured cell proliferation via an MTS assay. We were able to observe that an overexpression of Survivin–HA nearly completely rescued the antiproliferative effect of **L1** in all tested concentrations. While cell proliferation was reduced to 72 % (50 μm), 67 % (100 μm), and 55 % (200 μm) in control cells, cell viability could be restored to 97 % (50 μm), 89 % (100 μm), and 91 % (200 μm) in cells overexpressing Survivin–HA (Figure 7). These results suggest that the effects of **L1** are indeed caused by a specific inhibition of Survivin inside the cells.

**Figure 7:**
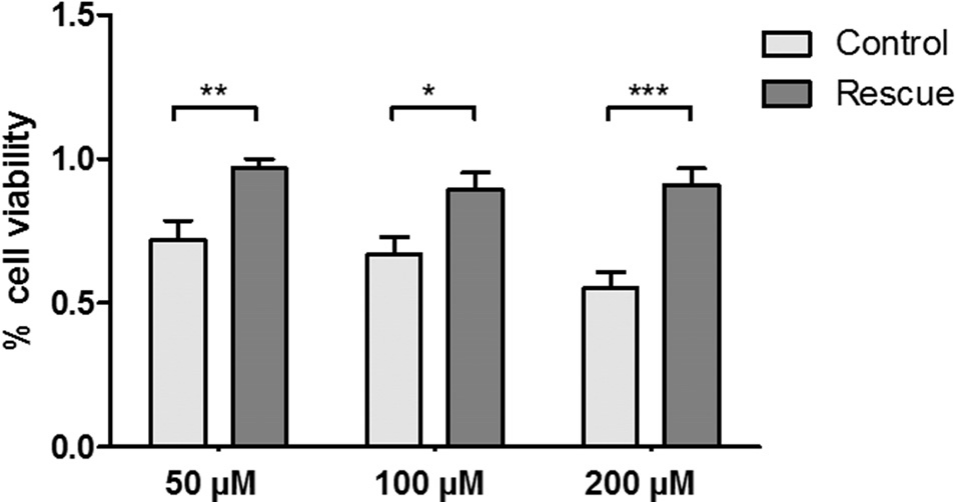
Overexpression of Survivin–HA rescues the antiproliferative effect of **L1** in HCT 116 cells and thereby confirms that the observed effects of the ligand are Survivin‐specific. HCT 116 were transiently transfected with Survivin–HA (Rescue) or GFP (Control) and treated with different concentrations of **L1** for 72 h. Cell proliferation was measured via an MTS assay. Experiments were performed in triplicate. The error bars show the standard error of the mean. Data was analyzed by a 1way ANOVA test followed by a Tukey's Multiple Comparison test. One asterisk (*) indicates a *p* value smaller than 0.05. Two asterisks (**) indicate a *p* value smaller than 0.01. Three asterisks (***) indicate a *p* value smaller than 0.001.

Based on a focused library we were able to identify **L1** as a potent ligand to target the cancer‐relevant protein Survivin by disrupting the protein–protein interaction with Histone H3. We verified binding of the ligand to Survivin's Histone H3 binding site, which mediates the interaction with Histone H3 in the early stages of mitosis and is crucial for cell proliferation. In addition, it was shown that the interaction between the two proteins was successfully decreased in a cellular environment. This resulted in an increasing number of mitotic defects and consequently in a reduction of cancer cell proliferation that was confirmed to be caused by a specific inhibition of Survivin inside the cells. Further studies now focus on the development of additional ligands in order to target other functionally relevant protein–protein interactions of Survivin. In addition, multivalency is explored as an approach to further improve and optimize **L1** regarding specificity and affinity.

## Conflict of interest

The authors declare no conflict of interest.

## Supporting information

As a service to our authors and readers, this journal provides supporting information supplied by the authors. Such materials are peer reviewed and may be re‐organized for online delivery, but are not copy‐edited or typeset. Technical support issues arising from supporting information (other than missing files) should be addressed to the authors.

Supplementary
