# Supplementary material for: Functional Disruption of the Cancer‐Relevant Interaction between Survivin and Histone H3 with a Guanidiniocarbonyl Pyrrole Ligand
Source: Angew Chem Int Ed Engl. 2020 Jan 28;59(14):5567–71. doi: 10.1002/anie.201915400 (PMC7155087; doi:10.1002/anie.201915400)
Supplement: Supplementary file 1 — Supplementary [file ANIE-59-5567-s001.pdf]

Angewandte Chemie
Eine Zeitschrift der Gesellschaft Deutscher Chemiker

Supporting Information

# Functional Disruption of the Cancer-Relevant Interaction between Survivin and Histone H3 with a Guanidiniocarbonyl Pyrrole Ligand

*Cecilia Vallet,* Dennis Aschmann, Christine Beuck, Matthias Killa, Annika Meiners, Marcel Mertel, Martin Ehlers, Peter Bayer, Carsten Schmuck†, Michael Giese,* and Shirley K. Knauer**

anie_201915400_sm_miscellaneous_information.pdf

**Table of Content**


1. General information S1
2. Synthesis S2
3. NMR titrations S7
4. Cultivation of eukaryotic cells S9
5. Co-immunoprecipitation S9
6. Proximity ligation assay S9
7. Immunofluorescence S10
8. Proliferation assay S11
9. NMR Spectra of synthesized compounds S11
10. Mass Spectra S17
11. HPLC Spectra S19
12. Mitotic defects in HeLa cells S20
13. Docking S21
14. References S25

## 1. General information

All solvents were distilled before use. Water was purified with a TKA MicroPure ultrapure water system. All other reagents were used as obtained from commercial sources unless otherwise specified. Reactions were monitored by TLC on silica gel plates (Macherey-Nagel POLYGRAM SIL G/UV254). Spots were visualized by UV light (254 nm and 366 nm). Lyophilisation was performed with a Christ Alpha 1-4 LDplus freeze dryer. The pH was determined with a pH-meter 766 Calimatic from Knick. The melting points were measured with a Büchi Melting-Point B-540 apparatus with open end glass capillary tubes. The melting points are not corrected. The NMR spectra of the synthesized compounds were recorded with Bruker DMX 300, DRX 500 or AVHD 600 spectrometers. All measurements were performed at room temperature using DMSO-$d_6$ as solvent. The chemical shifts are relative to the signals of DMSO-$d_6$ (δ $^1$H = 2.50 ppm and δ $^{13}$C = 39.5 ppm). The apparent coupling constants are given in Hertz. The description of the fine structure means: s = singlet, br. s = broad singlet, d = doublet, t = triplet, m = multiplet.

## 2. Synthesis

The GCP binding motif **D** was synthesized starting from Cbz-*L*-Asn-OH **A** and the synthesis of the introduced GCP **D** is literature known.[1]

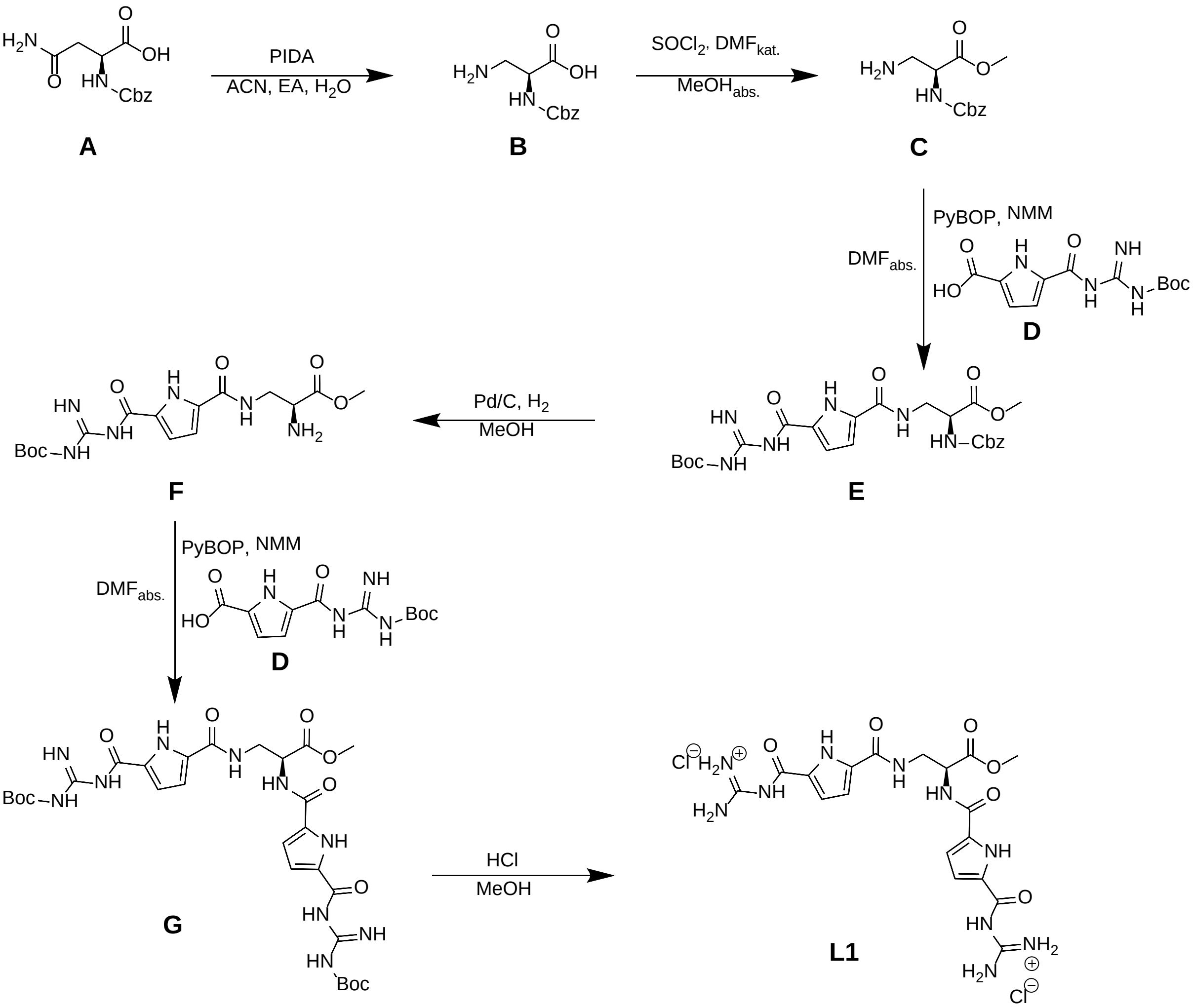


**Scheme S1:** Synthesis of Ligand **L1**.

### *L*-Dap(Cbz)-OH B

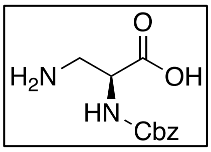

Cbz-*L*-Asn-OH **A** (10 g, 37.56 mmol) and (Diacetoxyiodo)benzene (15.28 g, 47.40 mmol) were dispersed in a mixture of Ethyl acetate (50 mL), Acetonitrile (50 mL) and $H_2O$ (25 mL) for 30 min at 0°C. The reaction was stirred over night at room temperature. The resulting dispersion was cooled at 0 °C and filtrated. After washing with Diethyl ether (20 mL) and drying in vacuo **B** (0.95 g, 3.79 mmol, 87%) was obtained as a colorless solid.

**Molecular Formular:** $C_{11}H_{14}N_2O_4$; **Molecular Mass:** 238.24 g/mol; **Mp:** 230.1 °C (decomposition); **$^1$H NMR:** (300 MHz, DMSO-$d_6$) δ [ppm] = 2.98-3.18 (m, 2H, ß-C$H_2$), 4.25 (m, 1H, α-C*H*), 5.04 (s, 2H, Cbz-C$H_2$), 7.35 (m, 5 H, Cbz-C*H*), 7.64 (d, 1H, $^3$J(H, H) = 7.95 Hz, α-N*H*), 7.84-9.60 (br. s, 2H, N$H_2$); **$^{13}$C NMR:** (75 MHz, DMSO-$d_6$) δ [ppm] = 51.8, 65.6, 127.7, 127.8, 128.3, 136.7, 156.0, 171.0; HR-MS: (pos. ESI, MeOH) m/z = 239.1030 ([M+H]+ , calc.: 239.1026).

### *L*-Dap(Cbz)-OMe C

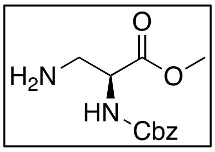

Cbz-*L*-Dap-OH **B** (1.1 g, 4.36 mmol) was dispersed in $MeOH_{abs.}$ (25 mL) and catalytic amounts DMF added, subsequently the reaction mixture was cooled to 0°C for 30 min, followed by slow and dropwise addition of $SOCl_2$ (0.65 mL, 8.72 mmol). The reaction mixture was stirred over night at 50 °C. The solvent was removed under reduced pressure and the residue recrystallized in MeOH/$Et_2O$ (1:1). *L*-Dap(Cbz)-OMe **C** (0.95 g, 3.79 mmol, 87%) was obtained as colorless crystalline needles.

**Molecular Formular:** $C_{12}H_{16}N_2O_4$; **Molecular Mass:** 252.27 g/mol; **Mp:** 170.5 °C; **$^1$H NMR:** (300 MHz, DMSO-$d_6$) δ [ppm] = 2.96-3.26 (m, 2H, C$H_2$), 4.43 (m, 1H, C*H*), 5.06 (s, 2H, Cbz-C$H_2$), 7.36 (m, 5H, Cbz-C*H*), 7.93 (d, 1H, $^3$J(H,H) = 8.15 Hz, N*H*), 8.30 (s, 3H, N$H_3$); **$^{13}$C NMR:** (75 MHz, DMSO-$d_6$) δ [ppm] = 51.7, 52.5, 65.8, 127.7, 127.9, 128.3, 136.6, 156.0, 169.8; HR-MS: (pos. ESI, MeOH) m/z = 253.1194 ([M+H]+ , calc.: 253.1183).

### GCP-*L*-Dap(Cbz)-OMe E

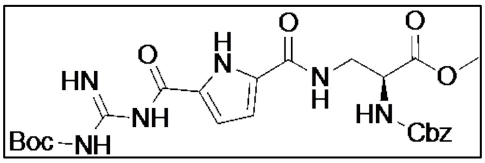

GCP **D** (6.0 g, 15.1 mmol) and PyBOP (9.42 g, 18.1 mmol) were dissolved in $DMF_{abs.}$ (50 mL), then NMM (10 mL, 90.9 mmol) was added and stirred at RT for 20 min. Subsequently, the free amine **C** (4.78 g, 16.6 mmol) was added and the mixture was stirred overnight. The reaction mixture was evaporated at reduced pressure and the residue was taken up with MeOH and precipitated in $H_2O$. The suspension was extracted three times with Ethyl acetate (100 mL), the organic layers were combined and washed with saturated $NaHCO_3$, $NaHSO_4$ (0.5 N) solution and water. The combined organic layers were dried with $MgSO_4$ and the solvent removed under reduced pressure. The crude product was purified by column chromatography ($SiO_2$, DCM/Acetone = 7/3) to give **E** (5.60 g, 10.6 mmol, 70%) as colorless solid.

**Molecular Formular:** $C_{24}H_{30}N_6O_8$; **Molecular Mass:** 530.54 g/mol; **Mp:** 103.1 °C (decomposition); $^1$H NMR: (300 MHz, DMSO-$d_6$) δ [ppm] = 1.45 (s, 9H, Boc-C$H_3$), 3.63 (m, 5H, β-C$H_2$ & C$H_3$), 4.30 (q, 1H, $^3$J(H,H) = 8.15 Hz, α-C*H*), 5.04 (s, 2H, Cbz-C$H_2$), 6.77 (m, 2H, Py-C*H*), 7.34 (m, 5H, Cbz-C*H*), 7.73 (d, 1H, $^3$J(H,H) = 7.89 Hz, N*H*), 8.48 (m, 2H, N*H*), 9.34 (s,

1H, N*H*), 9.34 (s, 1H, N*H*), 10.84 (s, 1H, N*H*), 11.36 (s, 1H, N*H*); **$^{13}$C NMR:** (75 MHz, DMSO-$d_6$) δ [ppm] = 27.7, 52.0, 53.8, 65.6, 112.0, 113.6, 127.7, 127.8, 128.3, 136.7, 155.9, 158.3, 160.1, 171.0; HR-MS: (pos. ESI, MeOH) m/z = 531.2251 ([M+H]+ , calc.: 531.2198), 553.2044 ([M+Na]+ , calc.: 553.2017).

**GCP-*L*-Dap-OMe F**

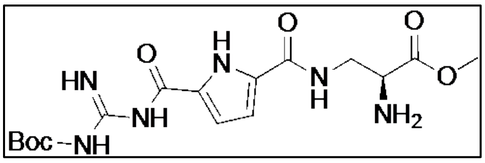


**E** (180mg, 0.34mmol) was dissolved in methanol (10mL), Pd/C (cat) was added and the mixture stirred at RT under $H_2$ atmosphere overnight. The mixture was filtered through a Celite pad, the solvent was removed under reduced pressure, and the resulting solid purified by MPLC on C18 reversed-phase silica gel (gradient 10% → 100% methanol/water in 60 min) to give **F** (95 mg, 0.24 mmol, 70%) as a white solid.

**Molecular Formular:** $C_{16}H_{24}N_6O_6$; **Molecular Mass:** 396.40 g/mol; **Mp:** 93.5 °C; **$^1$H NMR:** (300 MHz, DMSO-$d_6$) δ [ppm] = 1.45 (s, 9H, Boc-C*H*3), 3.32 (s, 2H, N*H2*), 3.40 (t, 1H, $^3J$(H,H) = 5.93 Hz, β-C*H2*), 3.51 (m, 1H, α-C*H*), 3.61 (s, 3H, C*H*3), 6.78 (m, 2H, Py-C*H*), 3.61 (t, 1H, $^3J$(H,H) = 5.82 Hz, N*H*), 8.57 (s, 1H, N*H*), 8.57 (s, 1H, N*H*), 9.32 (s, 1H, N*H*); **$^{13}$C NMR:** (75 MHz, DMSO-$d_6$) δ [ppm] = 27.7, 43.1, 51.6, 53.9, 112.0, 113.6, 158.3, 159.8, 174.5; HR-MS: (pos. ESI, MeOH) m/z = 397.1829 ([M+H]+ , calc.: 397.1830), 419.1646 ([M+Na]+, calc.: 419.1650).

**Protected Ligand G**

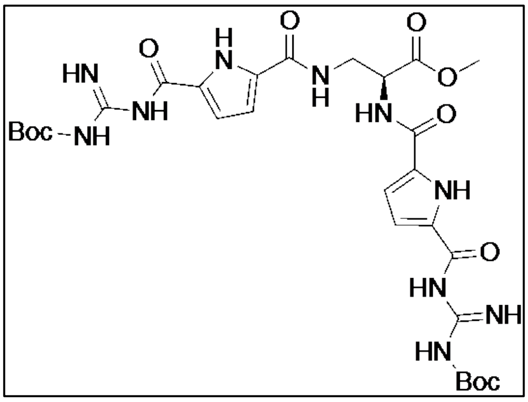


GCP **D** (12.68 g, 31.9 mmol) and PyBOP (19.61 g, 37.7 mmol) were dissolved in $DCM_{abs.}$ (50 mL), than NMM (19.13 mL, 0.17 mol) was added and stirred at RT for 20 min. Subsequently, the free amine **F** (11.5 g, 29.0 mmol) was added and the mixture was stirred overnight. The reaction mixture was evaporated at reduced pressure and the residue was taken up with MeOH and precipitated in $H_2O$. The suspension was filtered, the crude product was dried under reduced pressure and purified by column chromatography ($SiO_2$, DCM/Acetonitrile/MeOH = 9/1/0.5) to obtain **G** (16.04 g, 23.8 mmol, 82%) as colorless solid.

**Molecular Formular:** $C_{28}H_{38}N_{10}O_{10}$; **Molecular Mass:** 674.67 g/mol; **Mp:** 181 °C (decomposition); **$^1$H NMR:** (600 MHz, DMSO-$d_6$) δ [ppm] = 1.45 (s, 18H, Boc-C*H*3), 3.57 (m, 1H, ß-C*H2*), 3.64 (s, 3H, C*H*3), 3.76 (m, 1H, ß-C*H2*), 6.77 (m, 4H, Py-C*H*), 8.59 (m, 3H, N*H*), 8.81 (s, 1H, N*H*), 9.33 (br. s, 2H, N*H*), 10.83 (br. s, 4H, N*H*); **$^{13}$C NMR:** (151 MHz, DMSO-$d_6$) δ [ppm] = 27.8, 52.1, 112.3, 48.8, 112.7, 113.4, 158.4, 170.9; HR-MS: (pos. ESI, MeOH) m/z = 675.2853 ([M+H]+ , calc.: 675.2845).

**L1**

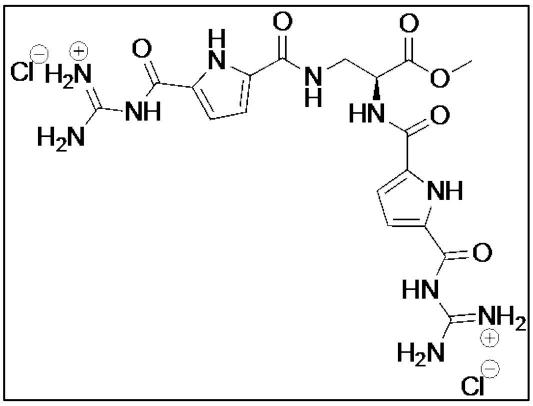

**G** (100 mg, 0.15 mmol) was dissolved in DCM (5 mL) followed by addition of TFA (3 mL). The reaction mixture was stirred for 4 h, then the solvent was removed under reduced pressure. The crude product was purified by MPLC on C18 reversed-phase silica gel (gradient 10% → 100% MeOH/$H_2O$ in 90 min, 0.1 % TFA) to give **L1** as TFA salt (93.5 mg, 0.13 mmol, 90%) as white solid. The hydrochloric salt **L1** was obtained as white solid, by addition of HCl (0.1 M, 2 mL) and lyophilization (three times).

**Molecular Formula:** $C_{18}H_{24}Cl_2N_{10}O_6$; **Molecular Mass:** 547.35 g/mol; **Mp:** 238 °C (decomposition); **$^1$H NMR**: (600 MHz, DMSO-$d_6$) δ [ppm] = 3.65 (m, 4H, β-C$H_2$ & C$H_3$), 3.77 (m, 1H, β-C$H_2$), 4.64 (q, 1H, $^3J$(H, H) = 6.18 Hz, C$H_2$), 6.87 (m, 2H, Py-C*H*), 7.42 (m, 2H, N*H*), 8.13-8.70 (m, 8H, N$H_2$), 12.25-12.73 (m, 2H, N*H*); **$^{13}$C NMR:** (151 MHz, DMSO-$d_6$) δ [ppm] = 52.2, 63.4, 112.7, 113.3, 115.8, 125.9, 131.9, 132.5, 155.4, 159.2, 159.6, 170.7; **HR-MS:** (pos. ESI, MeOH) m/z = 475.1806 ([M+H]+ , calc.: 475.1797), 497.1621 ([M+Na]+, calc.: 497.1616).

## 3. NMR titrations

### *Expression of $^{15}$N-labelled Survivin for NMR*

$^{15}$N-Survivin (1-120) was expressed as GST fusion protein with a PreScission Protease site in *E. coli* SoluBL21 (Genlantis) cells using M9 minimal media with $^{15}$N ammonium chloride as the sole nitrogen source. The protein was purified using a GSH affinity column. Subsequently, the GST tag was cleaved by addition of PreScission protease and the tagless $^{15}$N-Survivin was isolated using a Superdex S75 size exclusion column in tandem with a GSH affinity column to separate the Survivin dimer (27.7 kDa) from the free GST (28.0 kDa).

### *Protein NMR spectroscopy*

NMR experiments were recorded at 25°C on a Bruker 700 MHz Avance Ultrashield NMR spectrometer (Bruker, Germany) equipped with a 5 mm TCI $^{1}$H/$^{13}$C/$^{15}$N/D cryoprobe with z-gradient. The $^{1}$H,$^{15}$N-BEST-TROSY pulse sequence[2] is part of the NMRlib 2.0 pulse sequence tools from IBS (Grenoble, France; http://www.ibs.fr/research/scientific-output/software/pulse-sequence-tools/). Spectra were processed with Topspin 3.5 and analyzed in CARA[3]. The assignments of Survivin (1-120)[4] were obtained from the BMRB database (BMRB # 6342).

NMR samples were prepared in NMR buffer (50 mM $KP_i$ pH 6.5, 90 mM KCl, 2 mM DTT) containing 10% $D_2O$ with a protein concentration of 300 µM. A 10 mM stock of **L1** in DMSO-d6 was added stepwise, yielding a final ligand concentration of 300 µM in the presence of at most 4% DMSO-d6. $^{1}$H,$^{15}$N-BEST-TROSY spectra were recorded for each titration step. To account for slight shifting of signals due to the presence of DMSO, a control titration with the corresponding volumes of DMSO-d6 without ligand was performed.

The amide chemical shift perturbation $\Delta\delta_{total}$ was calculated from the $^{1}$H- and $^{15}$N-shifts according to equation 1[4] using the spectra with no and 300 µM of ligand. $\Delta\delta_N$ and $\Delta\delta_H$ represent the chemical shift perturbation value of the amide nitrogen and proton relative to the corresponding DMSO control spectrum without ligand:

$$\Delta\delta_{total} = \sqrt{\Delta\delta_H^2 + (0.154 \cdot \Delta\delta_N)^2} \qquad (1)$$

Furthermore, the relative intensities I/I0 were evaluated. A more than average decrease in intensity also indicates ligand binding due to intermediate exchange kinetics.

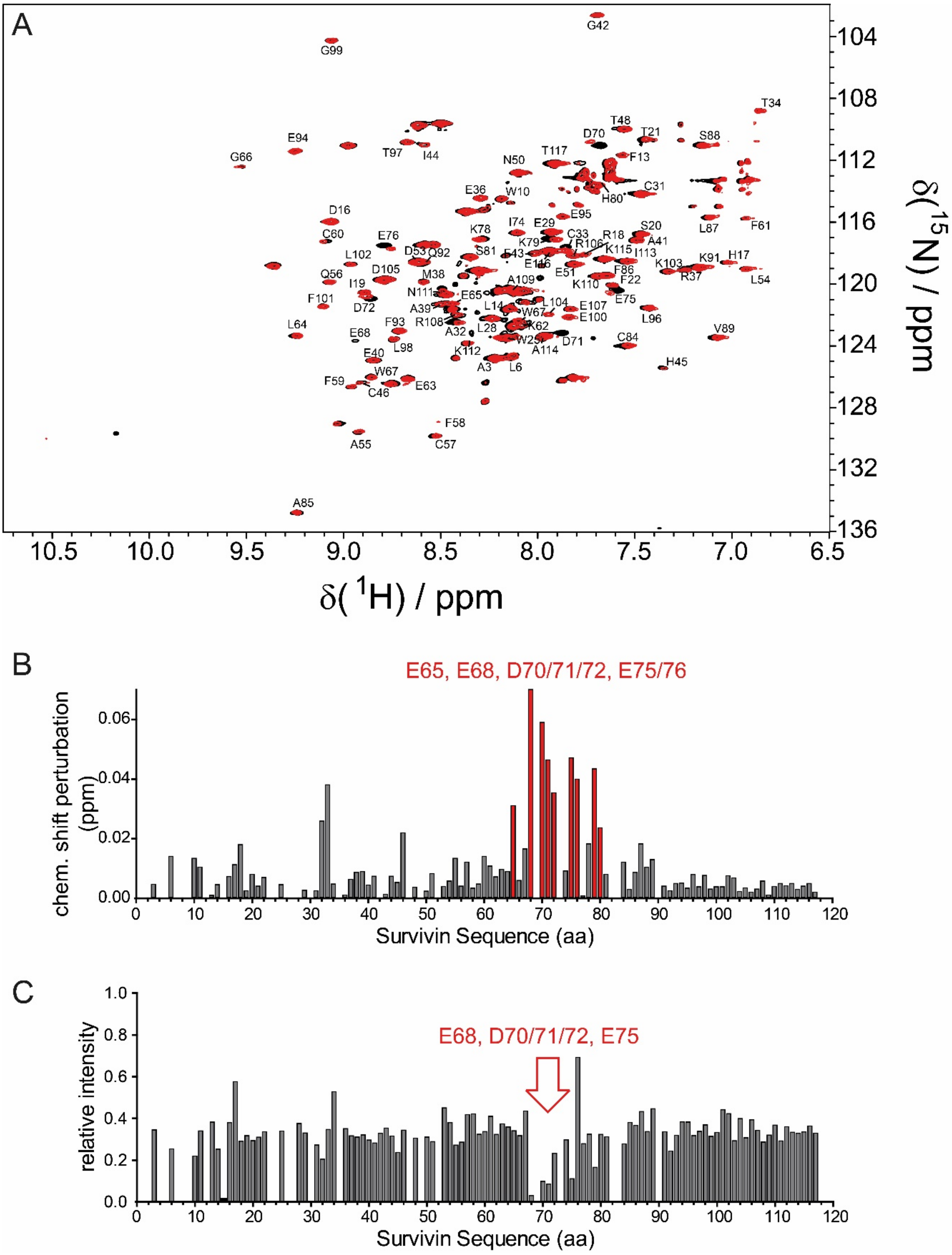


**Fig. S1:** (A) $^1$H-$^{15}$N-BEST-TROSY-HSQC spectra of 300 µM $^{15}$N-labeled Survivin 1-120 with (red) and without (black) 300 µM **L1**. (B) Chemical shift perturbations for each signal were plotted against the Survivin sequence. The residues with prominent chemical shift perturbations (> 0.02 ppm) are highlighted in red, amongst them the aspartic acid and glutamic acid residues within the Histone H3 binding site of Survivin. (C) The relative signal intensities show a more than average decrease within Survivin's Histone H3 binding site also indicating ligand binding within this region (red arrow).

## 4. Cultivation of eukaryotic cells

The adherent eukaryotic cell lines used in this work were cultivated in Dulbecco's Modified Eagle Medium (DMEM) supplemented with 10 % FCS and 1 % Antibiotic-Antimycotic (Life Technologies) at 37 °C, 5 % $CO_2$ and 90 % relative humidity. They were passaged twice a week in a ratio of 1:20 to regulate cell density and to supply the cells with fresh growth medium. For this, growth medium was aspirated and the cells were rinsed with 5 ml DPBS. Afterwards, 2 ml of TrypLE Express (Life Technologies) were added to the cells to allow enzymatic detachment from the cell culture flask. The cells were incubated on a heating plate until all of them were detached before 8 ml of new growth medium were added. 0.5 ml of the cell suspension were then added to a new culture flask together with 9.5 ml of fresh growth medium.

## 5. Co-immunoprecipitation

Co-immunoprecipitation was performed using the µMACS isolation kits (Miltenyi Biotec). Eukaryotic cells were transiently transfected with a plasmid coding for the HA-tagged Survivin. 24 h after transfection, cell lysates were generated by chemically lysing the cells with lysis buffer containing the detergents sodium deoxycholate and NP40. Cells were first detached from the 6 cm cell culture dish with a cell scraper. The cell suspension was then centrifuged at 500 x g for 5 min at 4 °C and washed with PBS before it was resuspended in 300 µl lysis buffer. After incubating the cells for 30 min on ice, they were sonicated twice with a Sonopuls mini20 ultrasonic homogenizer for 10 s and an amplitude of 90 %. After centrifugation at 20,000 x g for 20 min at 4 °C, the supernatant was transferred into a new reaction tube. Before adding 50 µl Tag antibody-coupled magnetic beads to the lysates, input samples were taken. The lysates were incubated on the beads for 1 h on ice and then transferred onto a µ column that had been placed into a µMACS separator and equilibrated with 200 µl interaction buffer. The column was washed four times with 200 µl lysis buffer and once with 100 µl wash buffer 2 from the kit. Elution was achieved by adding 20 µl preheated (95 °C) elution buffer from the kit onto the column and incubating for 5 min before adding another 50 µl of preheated elution buffer. The eluates were collected in 1.5 ml reaction tubes and input and eluate samples were analyzed by SDS-PAGE and Western Blot using antibodies against the HA-tag of Survivin-HA (901501, BioLegend) and Histone H3 (ab195277, abcam).

## 6. Proximity ligation assay

PLA staining was performed with the Duolink® In Situ Orange PLA Kit Mouse/Rabbit together with the Duolink® In Situ PLA® Probes and Detection Reagents from Sigma-Aldrich. Cells were seeded in 35 mm glass bottom dishes (MatTek) and fixed with 4 % Roti®-Histofix (Carl Roth) for 20 min at room temperature prior to PLA staining. Following three washing steps with PBS, the cells were permeabilized and unspecific binding sites were blocked for 30 min at 37 °C with blocking buffer containing TritonX-100 and normal goat serum. Afterwards, the cells were incubated at 4 °C over night with two primary antibodies derived from mouse (anti-Survivin, NB500-205, Novus Biologicals) and rabbit (anti-Histone H3 phospho T3, ab130940, abcam), which were diluted in antibody dilution buffer. Duolink® In Situ PLA probes Anti-Rabbit PLUS and Anti-Mouse MINUS were added after washing three times with PBS and incubated for 1 h

at 37°C. Following three additional washing steps, the ligation solution was incubated on the cells for 30 minutes at 37°C. After another three washing steps, the amplification reagents were added and incubated for 100 minutes at 37°C. Cells were then stained with 10 µg/ml Hoechst33342 and HCS CellMask™ Deep Red Stain (Thermo Scientific) in a dilution of 1:5000 in PBS for 20 min at RT in the dark before they were stored at 4 °C in 0.1% (w/v) sodium azide / PBS until they were microscopically analyzed. Confocal fluorescence microscopy images were taken with the scanning microscope TCS SP8 (Leica Microsystems) equipped with four lasers (Argon: 458/476/488/496/514 nm; DPSS: 561 nm; Helium Neon: 633 nm; UV Diode: 405 nm), two PMT confocal imaging detectors and one sensitive imaging hybrid detector. The samples were imaged with a HCX PL APO CS 63.0 x / 1.20 water objective or a HCX PL APO 63 x / 1.4–0.6 oil objective. The microscope was operated with the Leica Application Suite X (LAS X) software (Leica Microsystems). Subsequently, maximum projection images of z-stacks were analyzed with Cell Profiler. The outlines of the nuclei were defined based on Hoechst 33342 staining (primary objects) and the outlines of the entire cells were encircled based on Cell Mask staining and defined as secondary objects. PLA foci within the cells were then detected and assigned to the respective parental cells.

## 7. Immunofluorescence

Immunofluorescence is a technique to visualize proteins in cells with fluorophore-conjugated antibodies. In this work, the indirect immunofluorescence (IF) staining was applied, where two different antibodies, a target specific primary antibody (anti-Centromere, NB500-101, Novus Biologicals; anti-alpha-Tubulin, T6074, Sigma-Aldrich) and a fluorophore-conjugated secondary antibody (anti-human AF568, A21090, Invitrogen; anti-mouse AF488, A11001, Invitrogen), were used. Cells were seeded in culture dishes suitable for microscopy and prior to IF staining fixed with 4 % Roti®-Histofix (Carl Roth) for 20 min at RT. Following three washing steps with PBS, the cells were permeabilized and unspecific binding sites were blocked for 30 min at 37 °C with blocking buffer containing TritonX-100 and normal goat serum. Afterwards, the cells were incubated with primary antibodies diluted in antibody dilution buffer containing TritonX-100 and BSA at 4 °C over night. After washing three times with PBS, the secondary antibody solution was added onto the cells and incubated for 1 h at room temperature in the dark. The secondary antibody was again diluted in antibody dilution buffer and was supplemented with 10 µg/ml Hoechst33342. Following three final washing steps with PBS, the samples were stored at 4 °C in 0.1% (w/v) sodium azide/PBS before they were analyzed with the confocal fluorescence microscope. Confocal fluorescence microscopy images were taken with the scanning microscope TCS SP8 (Leica Microsystems) equipped with four lasers (Argon: 458/476/488/496/514 nm; DPSS: 561 nm; Helium Neon: 633 nm; UV Diode: 405 nm), two PMT confocal imaging detectors and one sensitive imaging hybrid detector. The samples were imaged with a HCX PL APO CS 63.0 x / 1.20 water objective or a HCX PL APO 63 x / 1.4–0.6 oil objective. The microscope was operated with the Leica Application Suite X (LAS X) software (Leica Microsystems). Data was analyzed via t test using a 99% confidence interval.

## 8. Proliferation assay

The CellTiter 96® AQueous One Cell Proliferation Assay (Promega) was used to observe cell proliferation after treatment with different ligand concentrations. The assay determines the number of viable cells per well by using the tetrazolium compound [3-(4,5-dimethylthiazol-2-yl)-5-(3-carboxymethoxyphenyl)-2-(4-sulfophe-nyl)-2H-tetrazolium (MTS) that is bioreduced by NADPH or NADH in living cells into a colored formazan product. Cells were seeded into a black 96-Well glass bottom dish (Corning) and incubated with different ligand concentrations for 72 h (triplicates for each condition). The assay was performed according to the manufacturer's protocol by adding 20 µl of CellTiter 96® AQueous One Solution Reagent directly into each culture well and incubating for 4 h. Afterwards, the absorption was measured at 490 nm with a GloMax®-Multi plate reader (Promega).

## 9. NMR Spectra of synthesized compounds

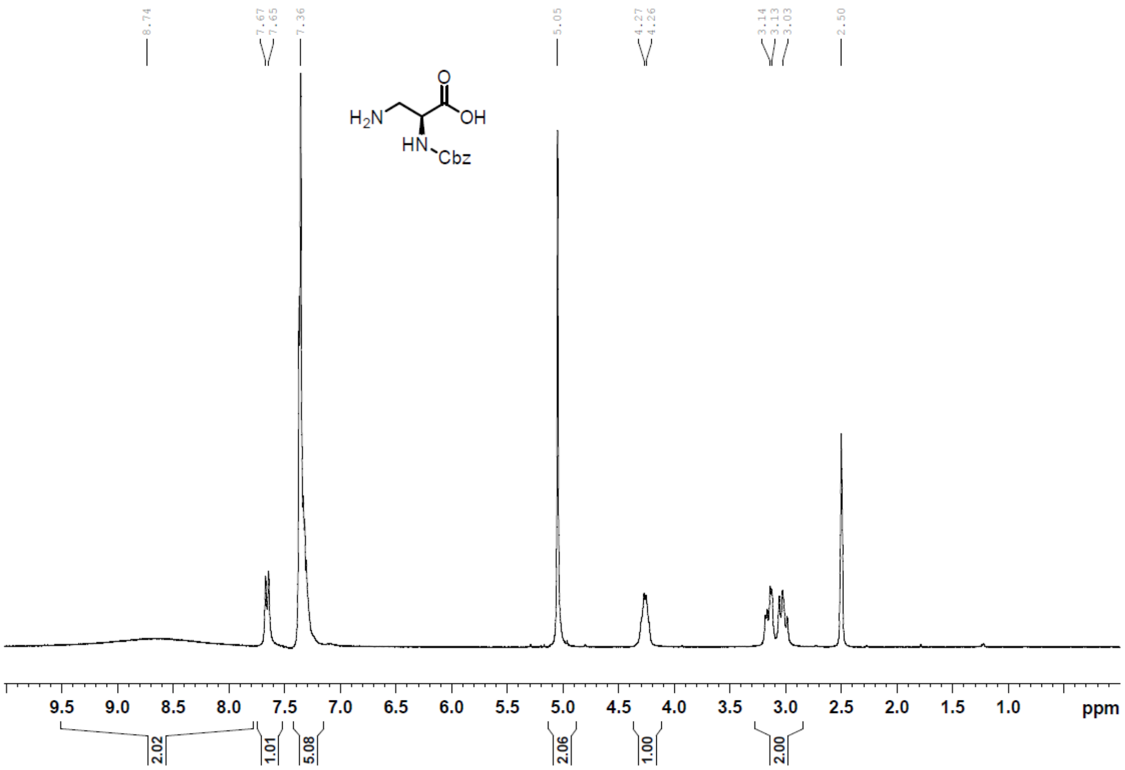


**Fig. S4:** $^{1}$H-NMR spectrum of **B** (600 MHz, DMSO-$d_6$).

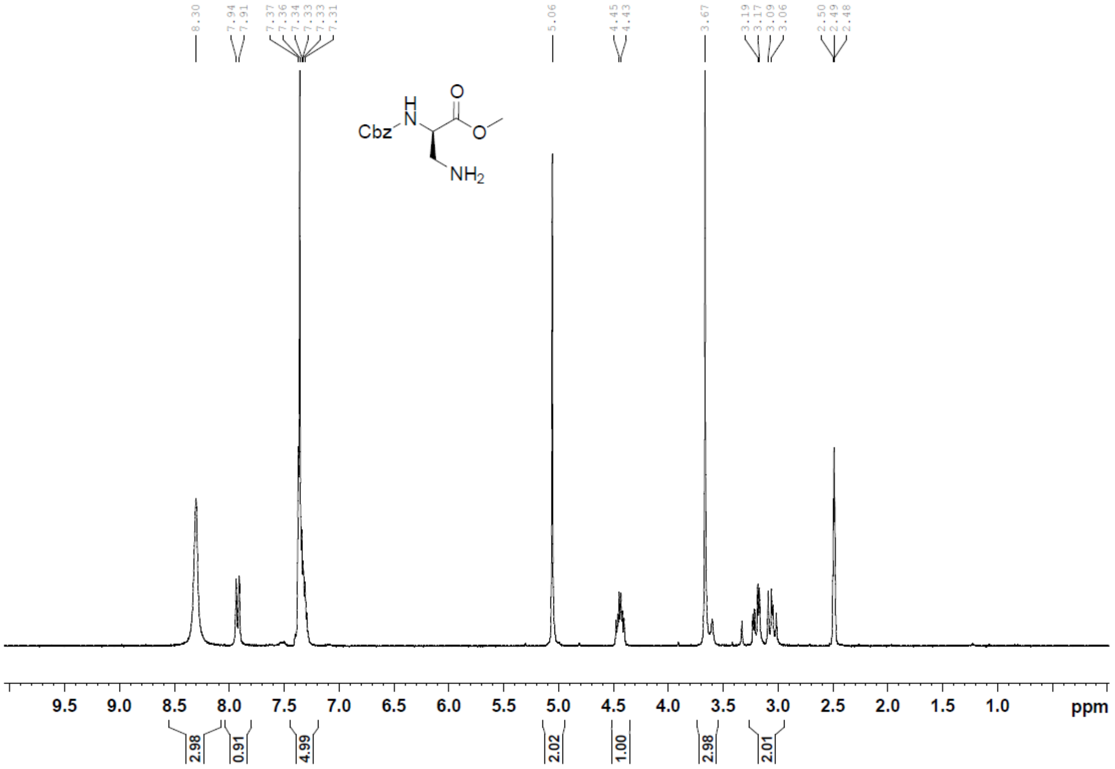


**Fig. S5:** $^{1}$H-NMR spectrum of **C** (300 MHz, DMSO-$d_6$).

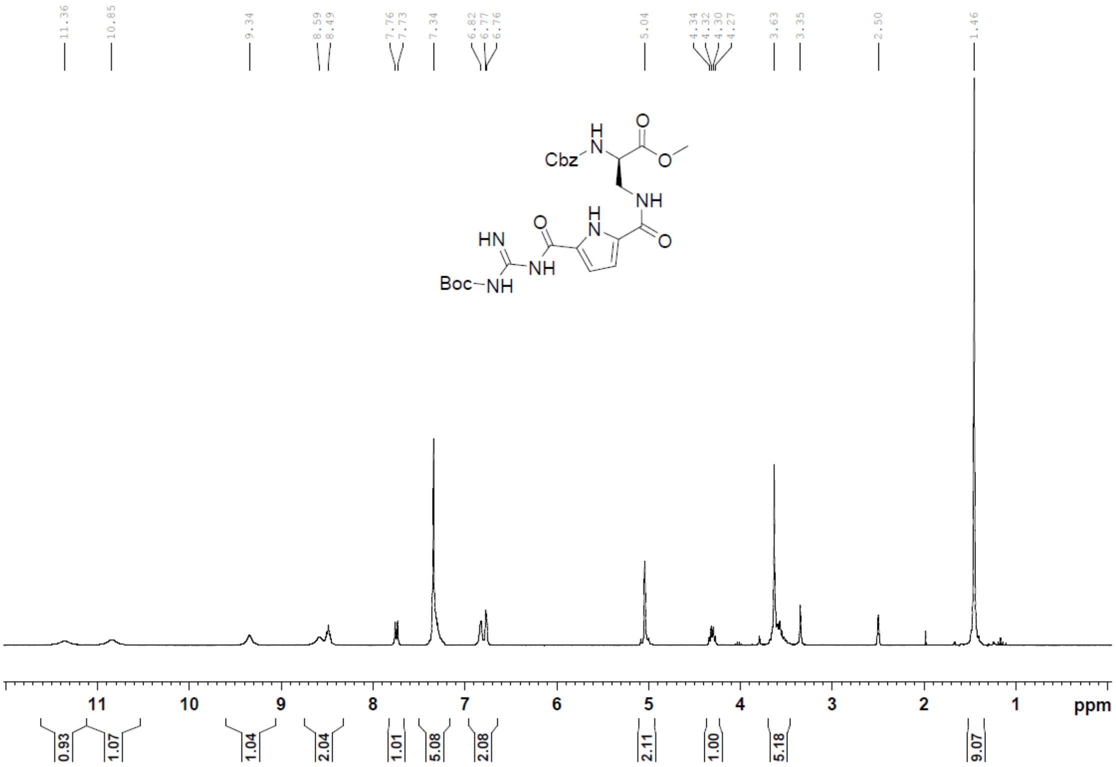


**Fig. S6:** $^{1}$H-NMR spectrum of **E** (300 MHz, DMSO-$d_6$).

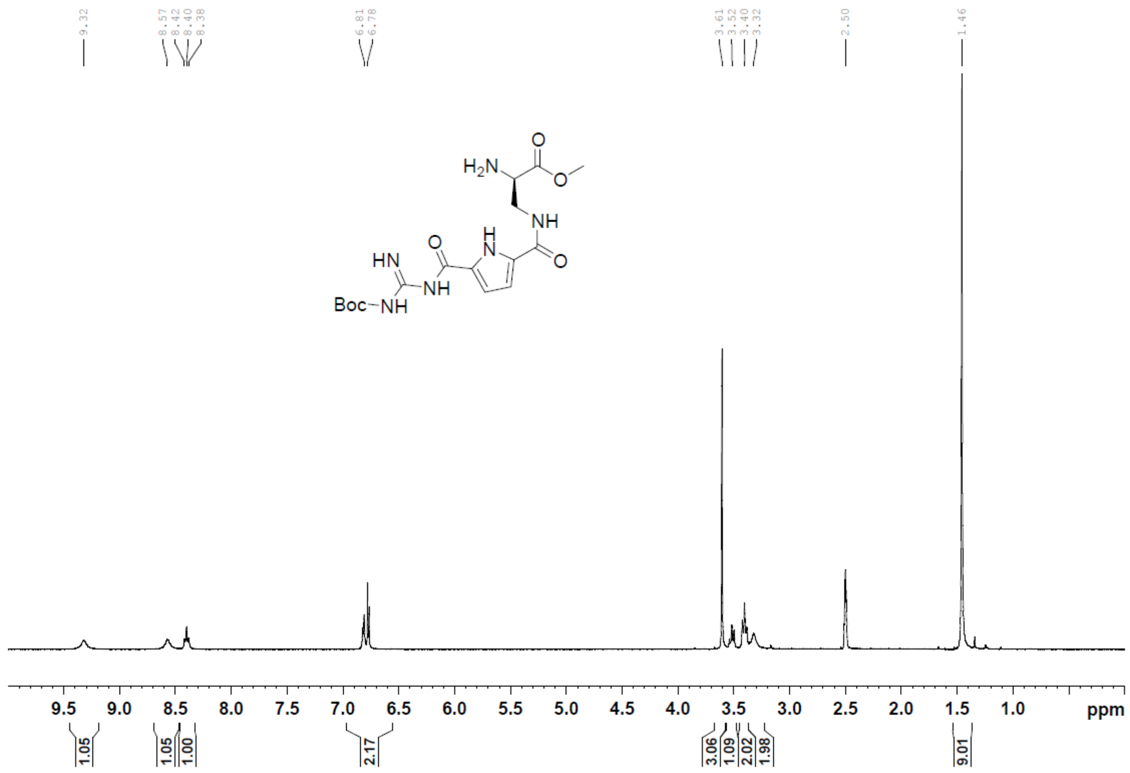


**Fig. S7:** $^{1}$H-NMR spectrum of **F** (300 MHz, DMSO-*d*$_6$).

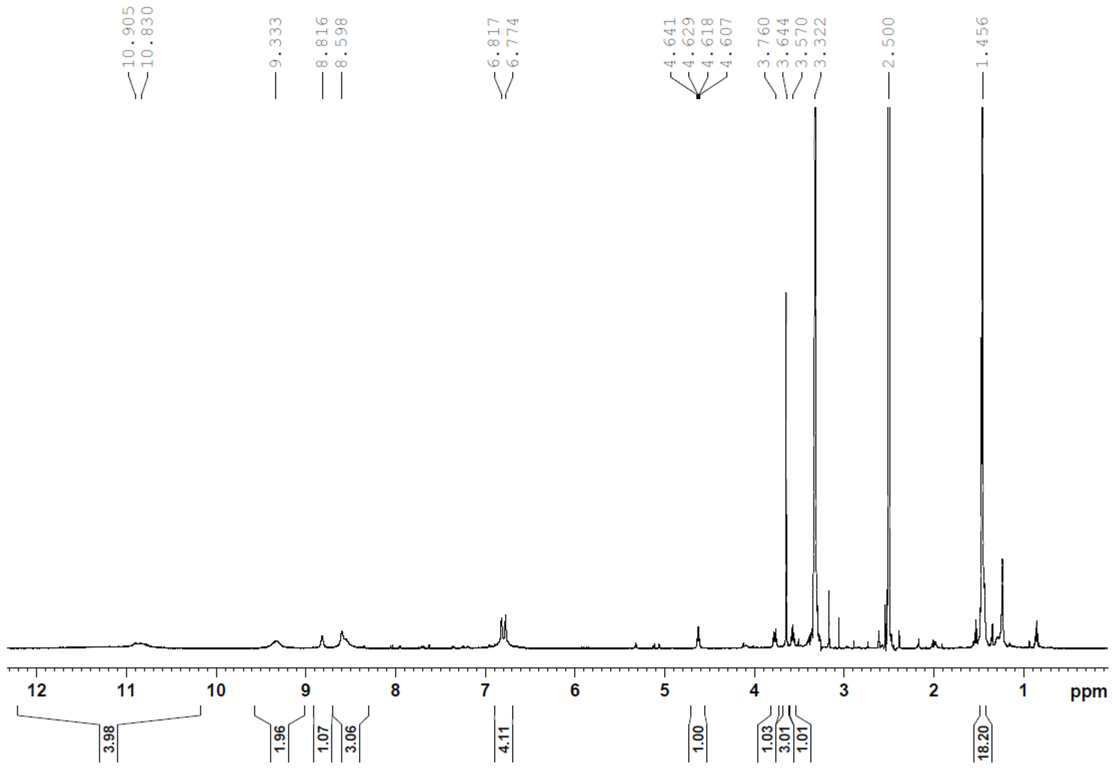


**Fig. S8:** $^{1}$H-NMR spectrum of **G** (600 MHz, DMSO-*d*$_6$).

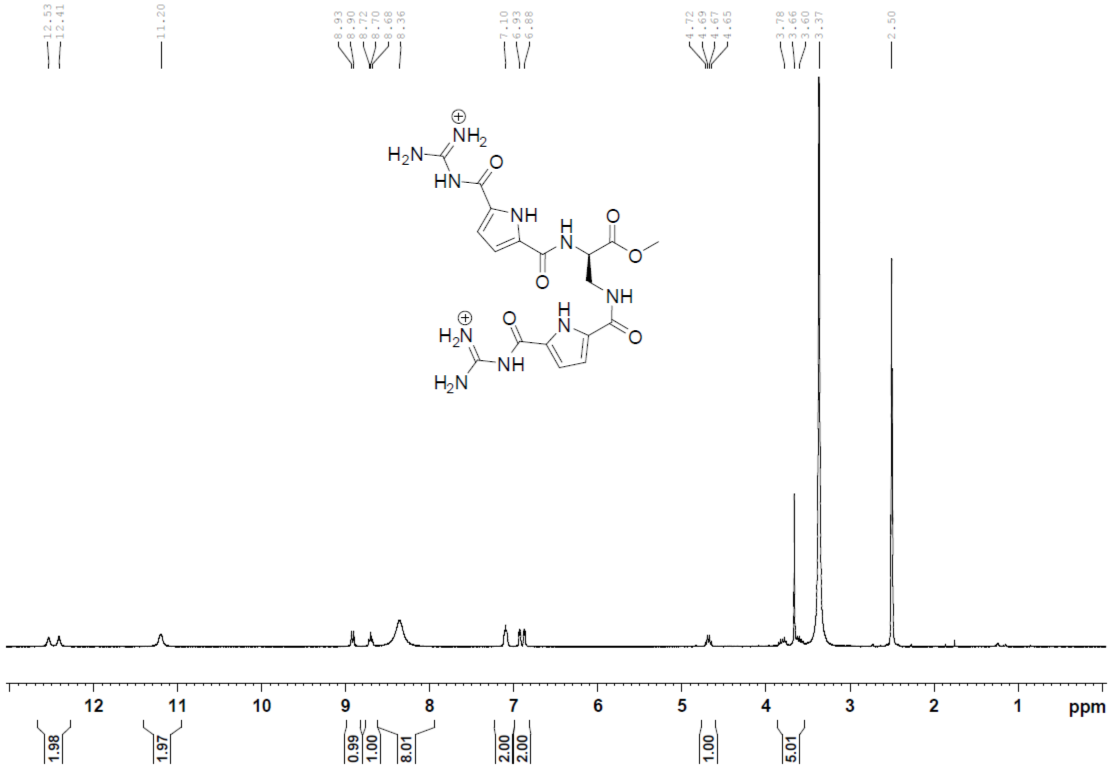


**Fig. S9:** $^{1}$H-NMR spectrum of **L1** (600 MHz, DMSO-$d_6$).

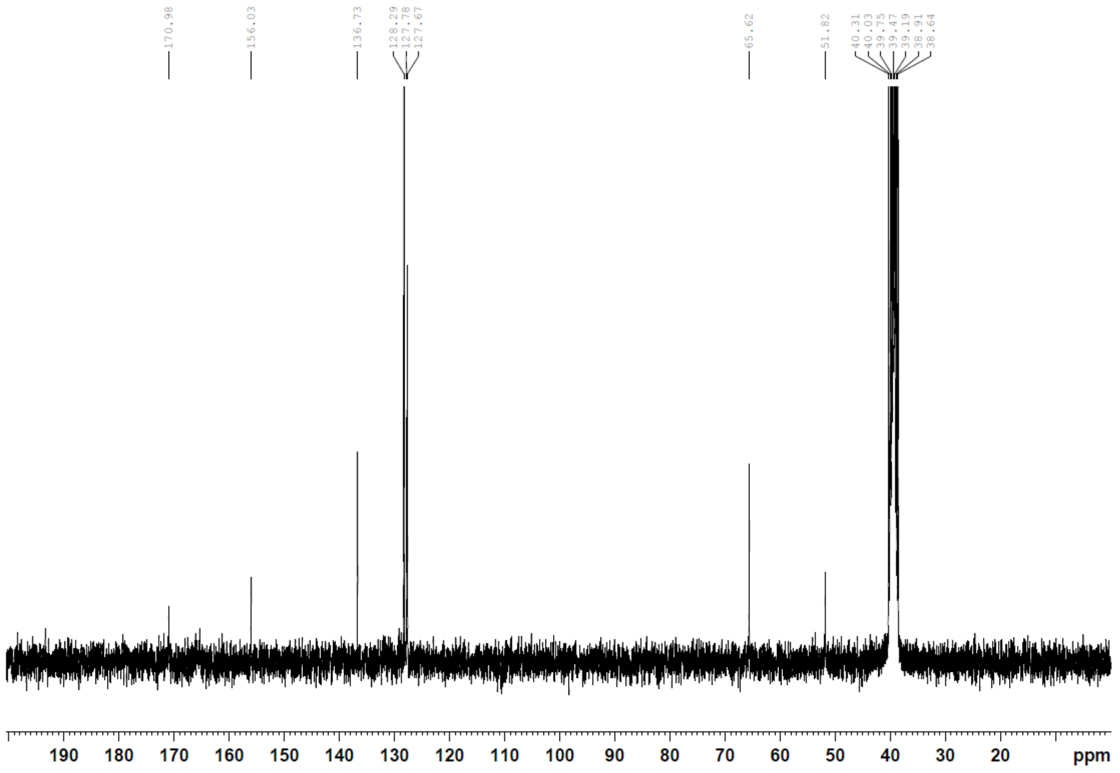


**Fig. S10:** $^{13}$C-NMR spectrum of **B** (75 MHz, DMSO-$d_6$).

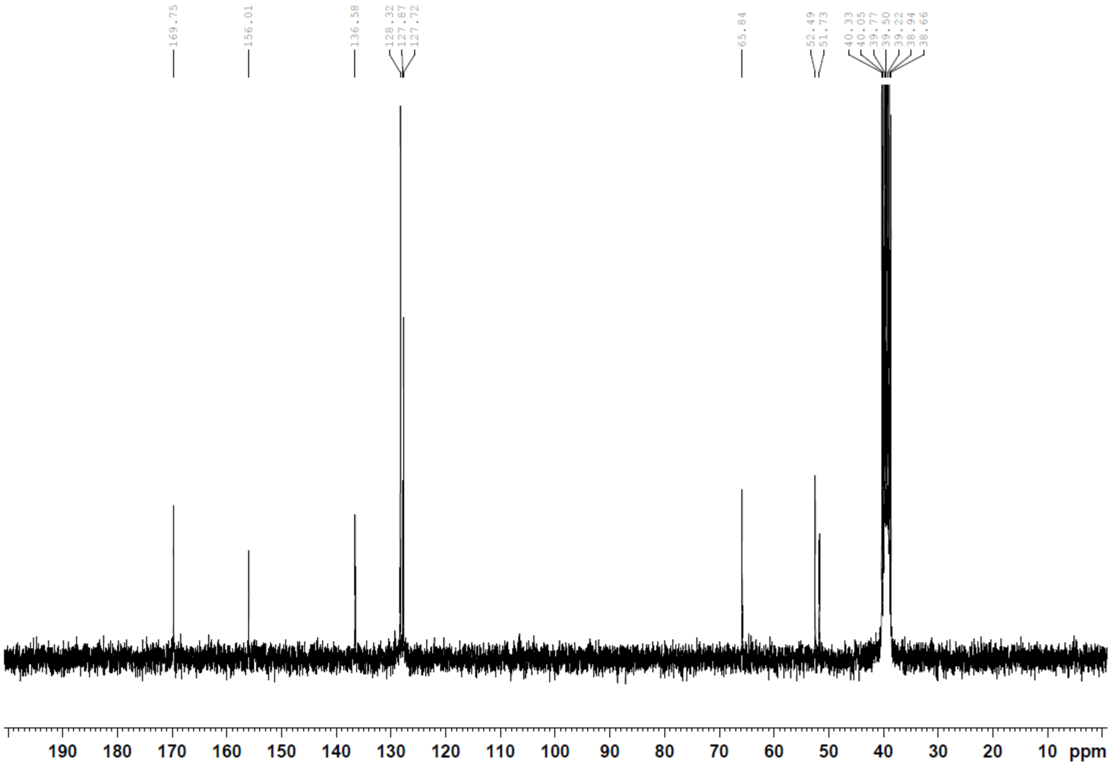


**Fig. S11:** $^{13}$C-NMR spectrum of **C** (75 MHz, DMSO-$d_6$).

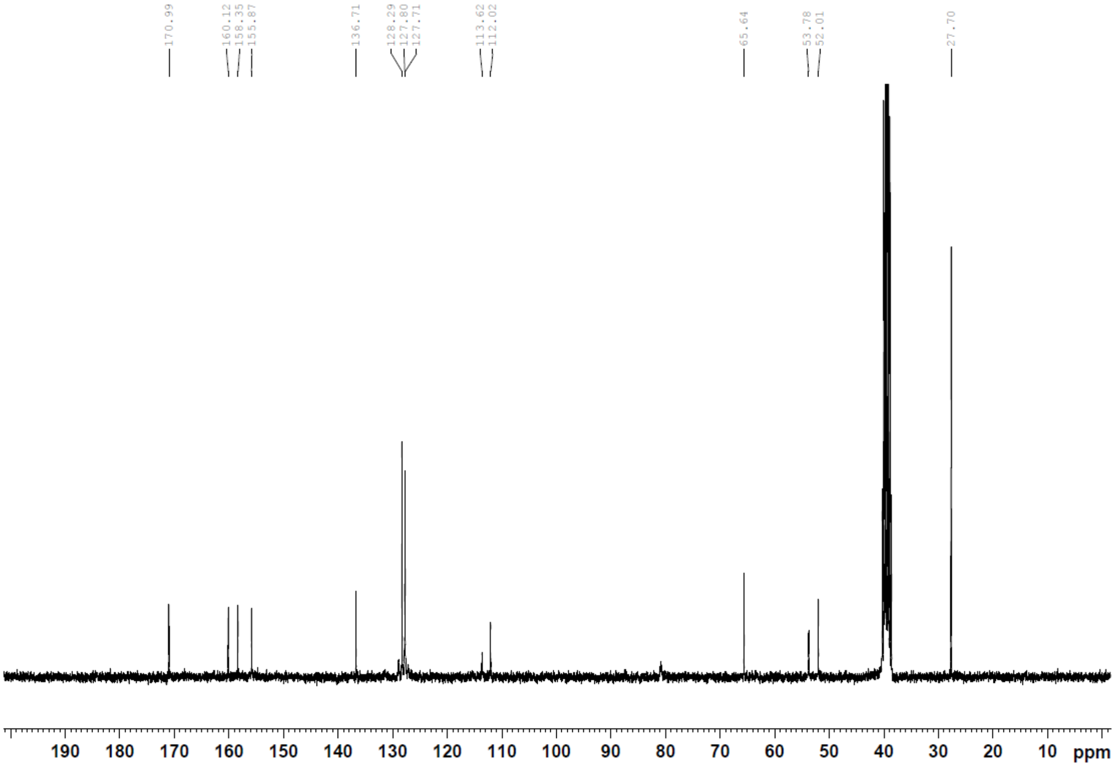


**Fig. S12:** $^{13}$C-NMR spectrum of **E** (75 MHz, DMSO-$d_6$).

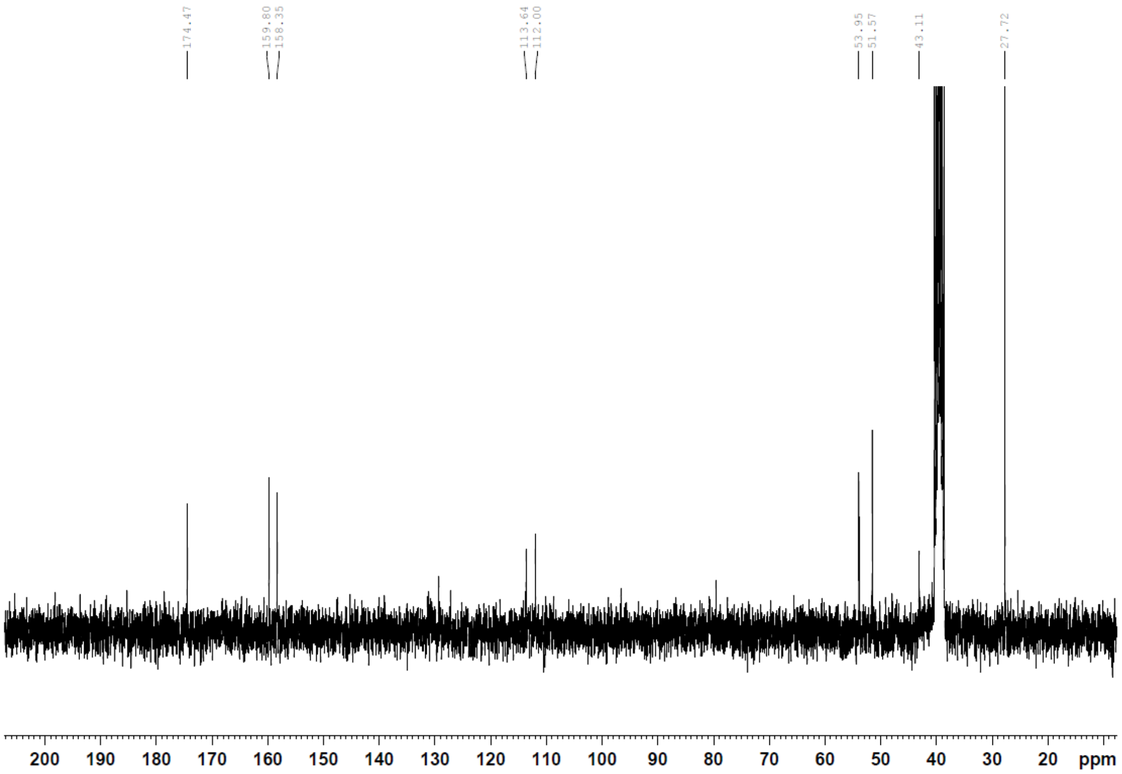


**Fig. S13:** $^{13}$C-NMR spectrum of **F** (75 MHz, DMSO-$d_6$).

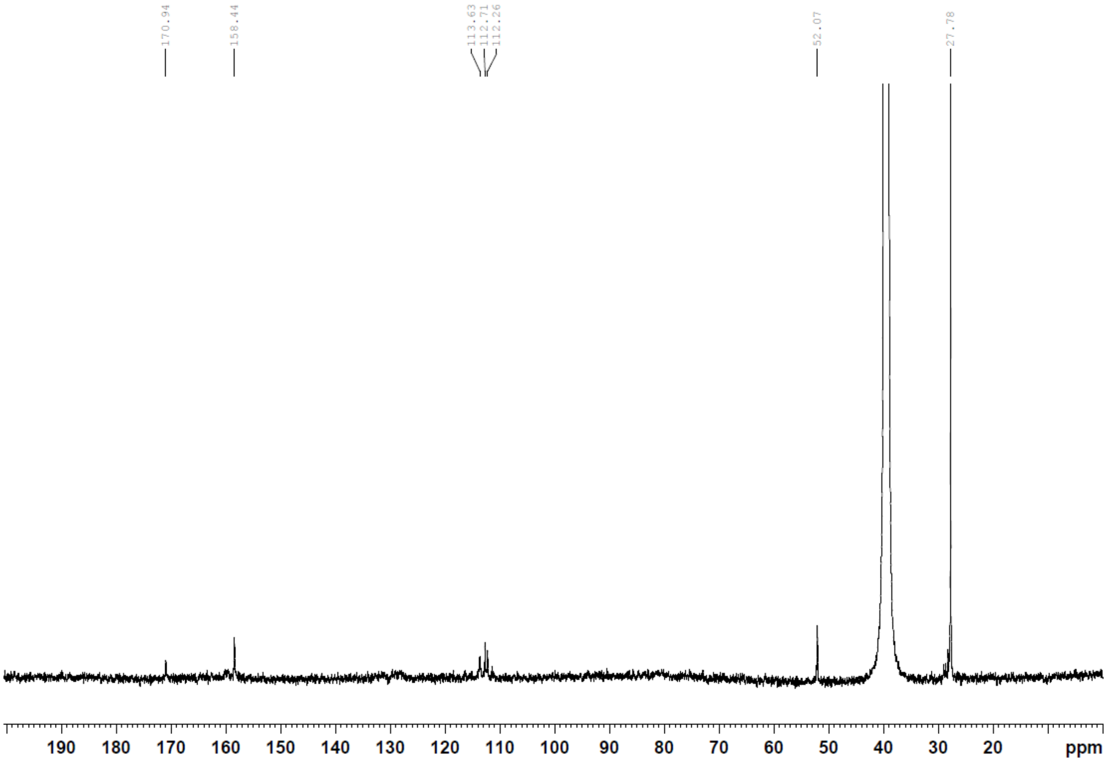


**Fig. S14:** $^{13}$C-NMR spectrum of **G** (151 MHz, DMSO-$d_6$).

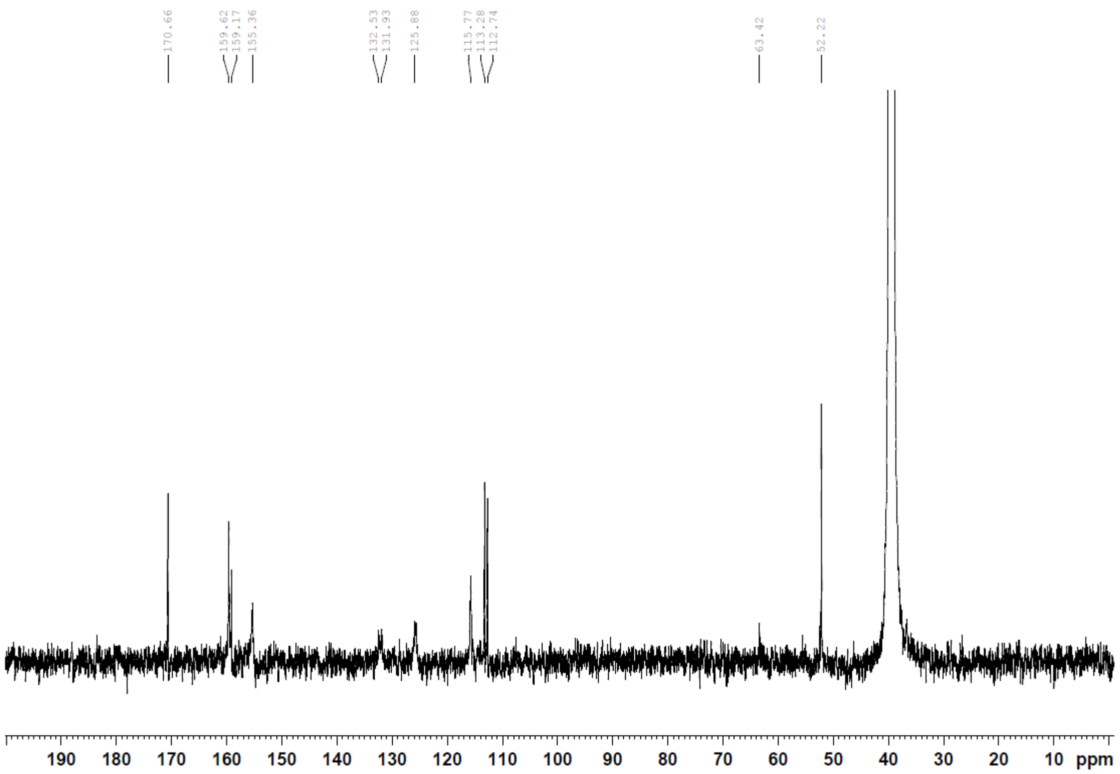


**Fig. S15:** $^{13}$C-NMR spectrum of **L1**(151 MHz, DMSO-$d_6$).

## 10. Mass Spectra

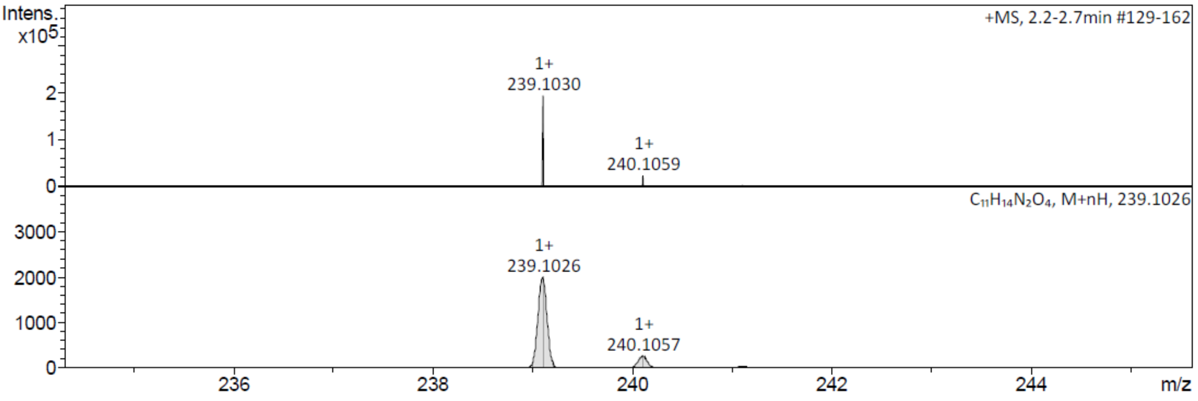


**Fig. S16:** HR-ESI mass spectrum of **B** (positive ion mode, MeOH) and predicted mass spectrum of peaks which belongs to **B.**

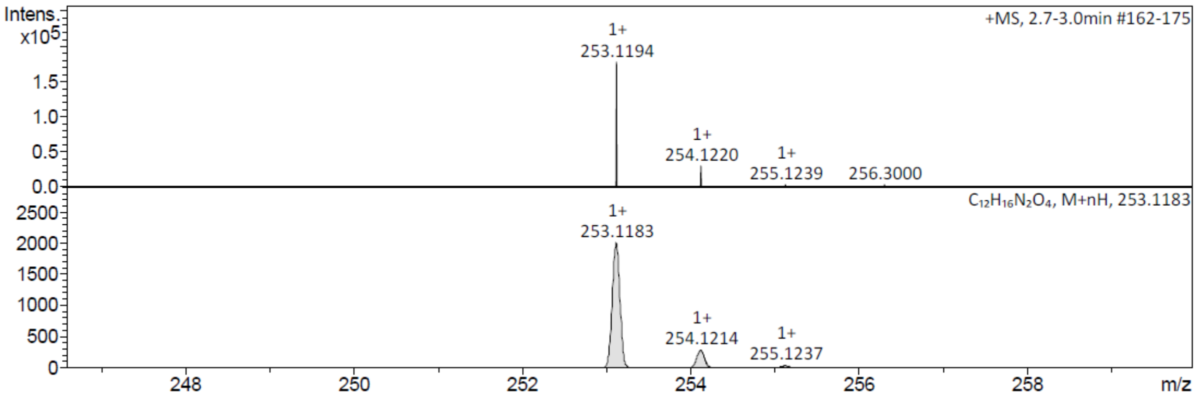


**Fig. S17:** HR-ESI mass spectrum of **C** (positive ion mode, MeOH) and predicted mass spectrum of peaks which belongs to **C.**

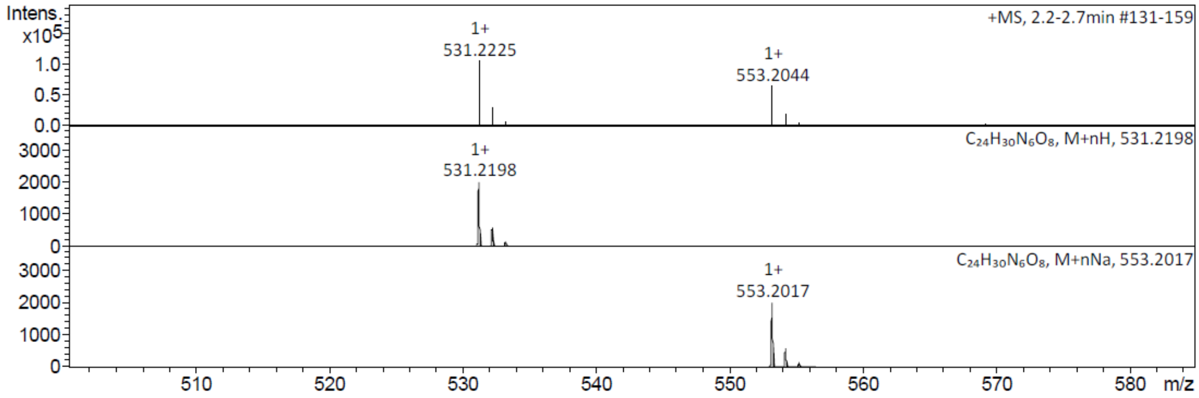


**Fig. S18:** HR-ESI mass spectrum of **D** (positive ion mode, MeOH) and predicted mass spectrum of peaks which belongs to **D.**

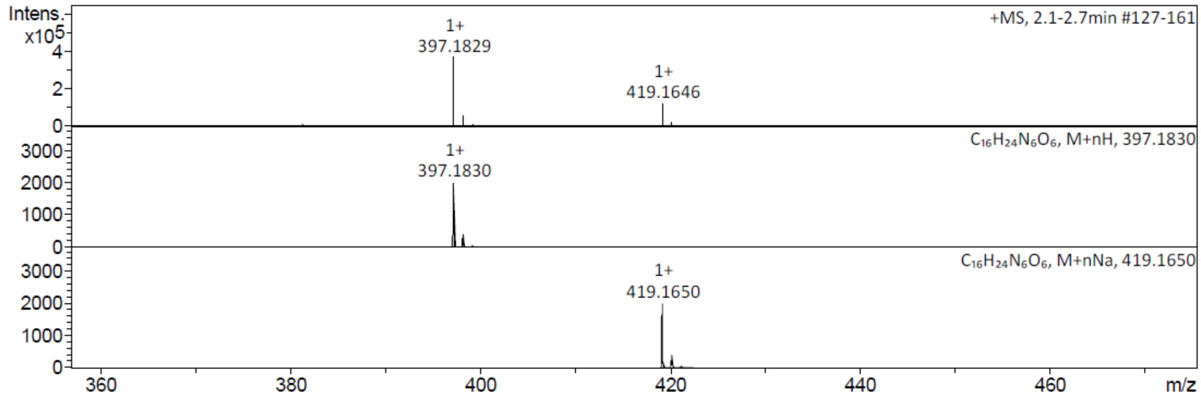


**Fig. S19:** HR-ESI mass spectrum of **F** (positive ion mode, MeOH) and predicted mass spectrum of peaks which belongs to **F.**

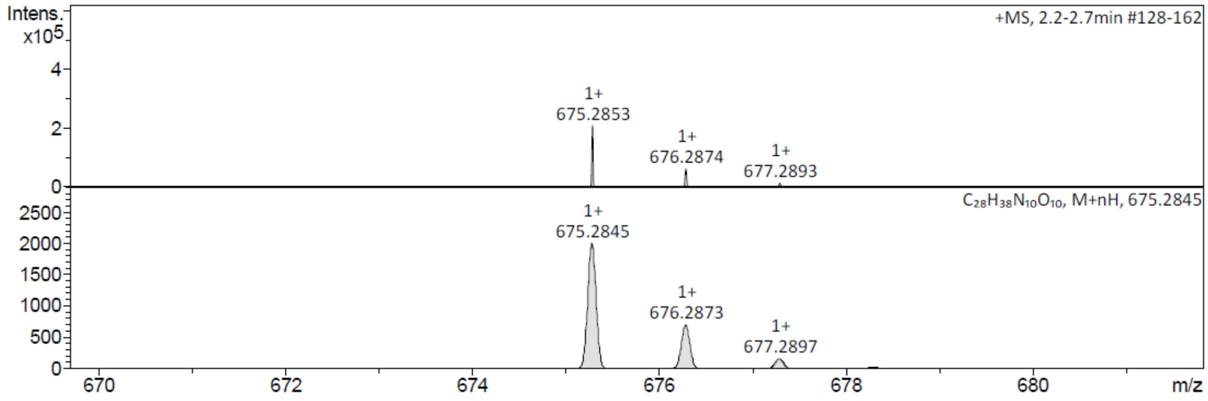


**Fig. S20:** HR-ESI mass spectrum of **G** (positive ion mode, MeOH) and predicted mass spectrum of peaks which belongs to **G.**

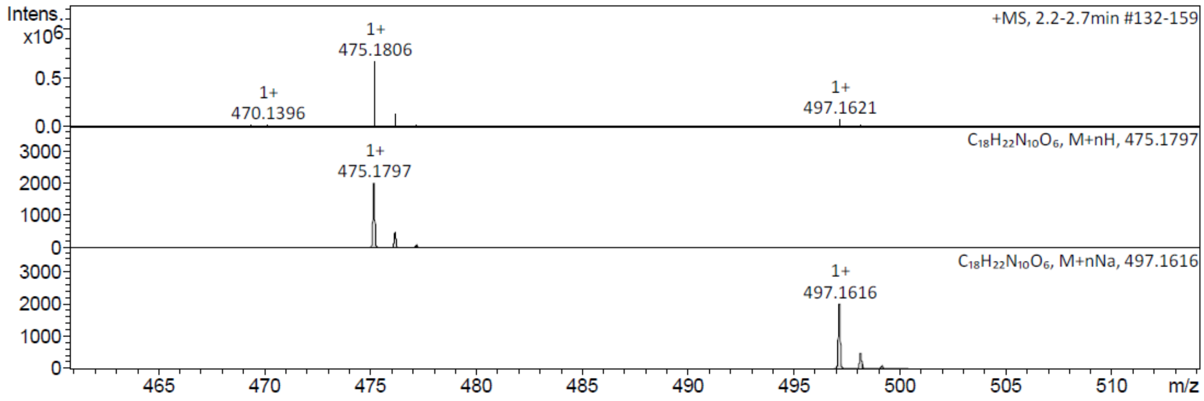


**Fig. S21:** HR-ESI mass spectrum of **L1** (positive ion mode, MeOH) and predicted mass spectrum of peaks which belongs to **L1.**

## 11. HPLC Chromatogram & UV Spectrum

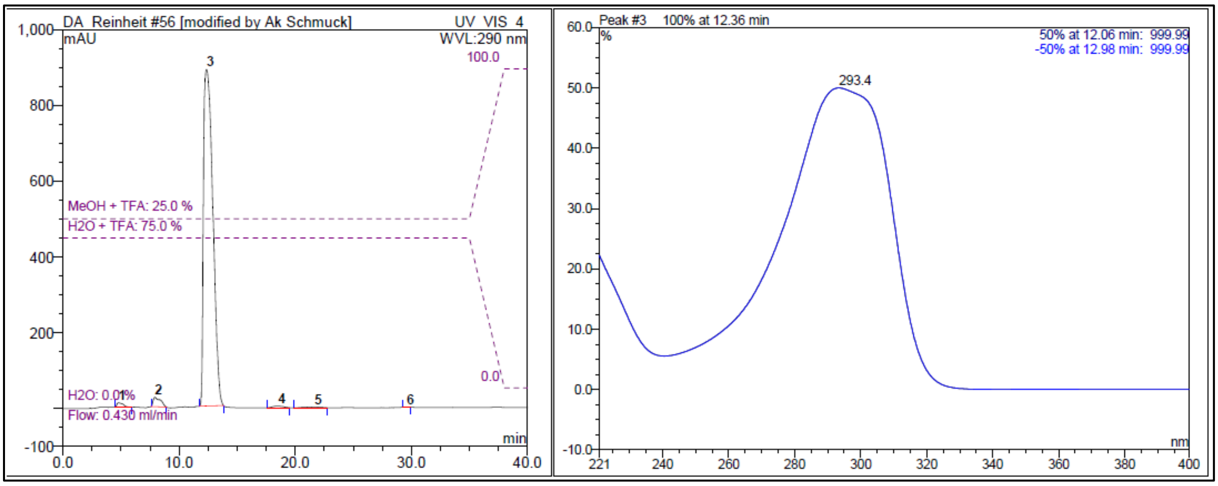


**Fig. S22:** HPLC analysis of **L1** isocratic: 25% MeOH/$H_2O$/TFA (0.1%) in 40 min, flow rate: 0.43 ml/min, retention time $t_R$ = 12.36 min, purity: >96% (peak integration based on UV-detection at 290 nm).

## 12. Mitotic defects in HeLa cells

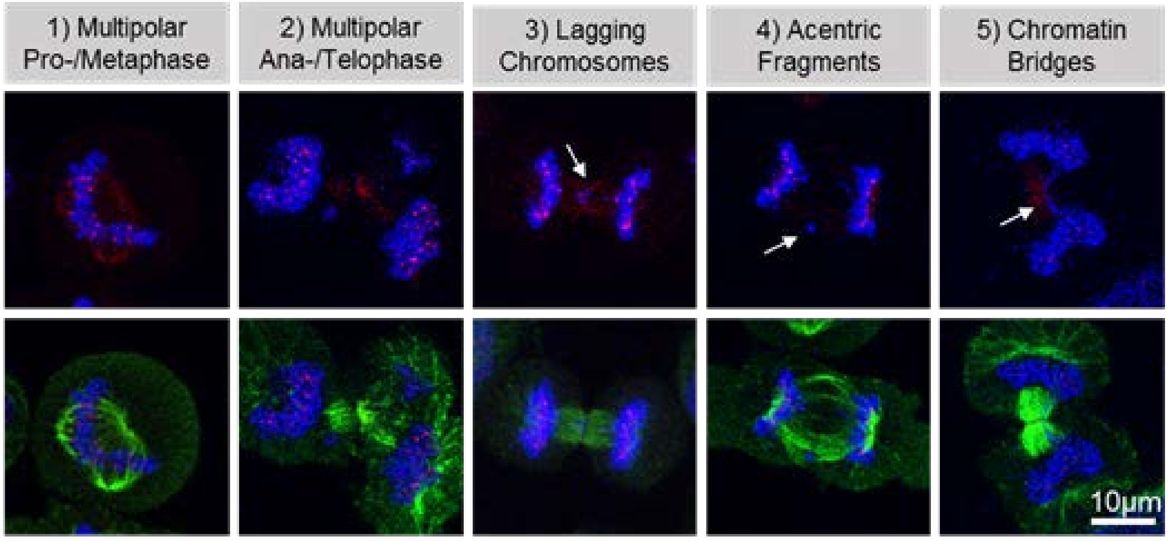


**Fig. S23:** Types of mitotic defects found in HeLa cells treated with 50 μM **L1** for 48 h and synchronized with RO-3306. Cells were fixed and immunostained. DNA is shown in blue, centromeres in red and α-Tubulin in green. White arrows highlight chromosome segregation defects.

1) Multipolar Prometa-/Metaphases are cells during prometaphase or metaphase with more than two spindle poles.

2) Multipolar Ana-/Telophases describe cells with more than two spindle poles in which the chromosomes might be segregating in more than two groups.

3) Lagging Chromosomes stay behind at the spindle midzone, while the other chromosomes move towards the spindle poles. In contrast to acentric fragments, they possess a centrosome.

4) Acentric Fragments appear as DNA fragments that are separated from the rest of the chromosomes but lack a centrosome.

5) Chromatin Bridges exhibit the feature of a “DNA bridge” between the two groups of segregating chromosomes. They can be connected to both groups of segregating chromosomes or disconnected on one or both ends.

## 13. Docking

The program Maestro 11.5 Schroedinger was used for docking experiments. 15 GCP aminoacid ligands were used for Ligand Preparation (LigPrep). Tautomers and possible states at pH=7 +/-2 were generated.

GCParg

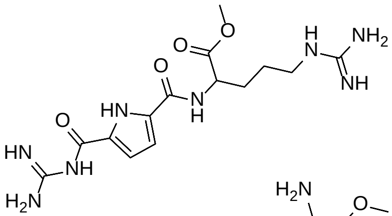

lysGCP

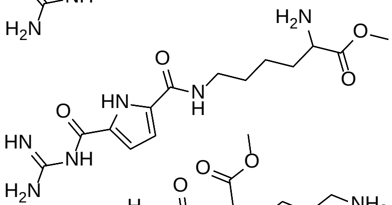

GCPlys

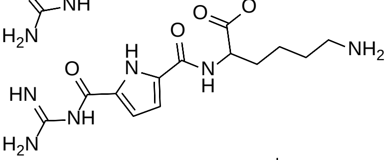

GCPglu

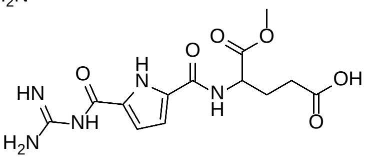

GCPphe

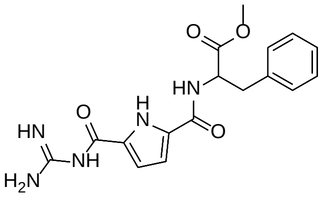

GCPleu

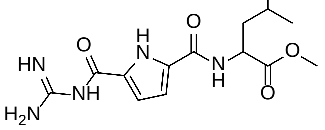

GCPgly

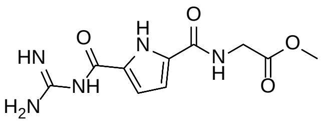

GCPAala
Aminoalanine

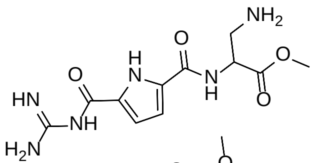

GCPasn

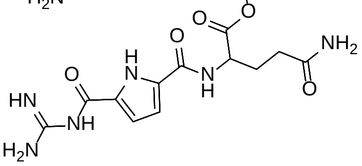

GCPAalaGCP

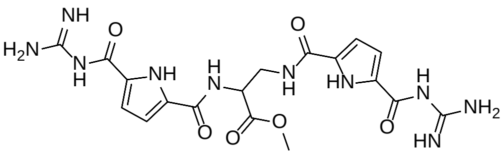

GCPAalaGCPBz

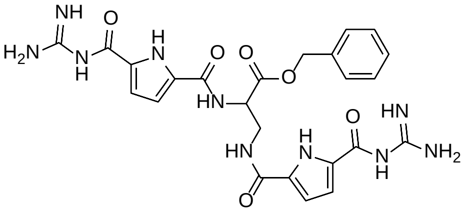

GCPAalaGCPtBu

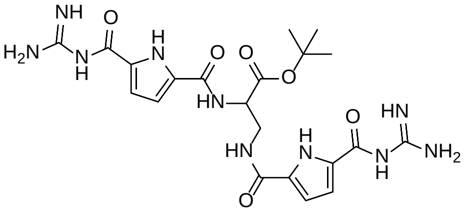

argarg

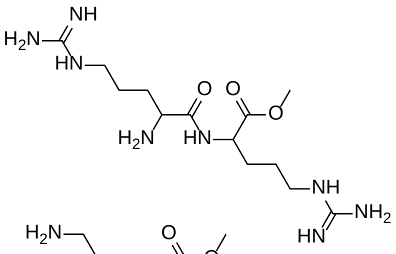

lyslys

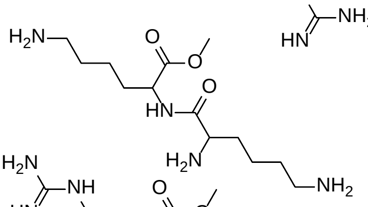

lysarg

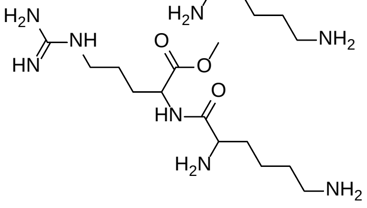

The protein structure of Survivin (PDB 1XOX) was prepared (Protein Preparation). Hydrogens were added. Zero-order bonds to metals and disulfide bonds were created. Missing chains and loops were added. Water beyond 5 Å from het groups was deleted. Het states were generated by using Epik (pH=7 +/- 2)

A grid around the amino acids GLU 68, ASP 70 and GLU 75 with a size of 25 Å was generated (Glide Grid Generation). All ASP and GLU were colored in red.

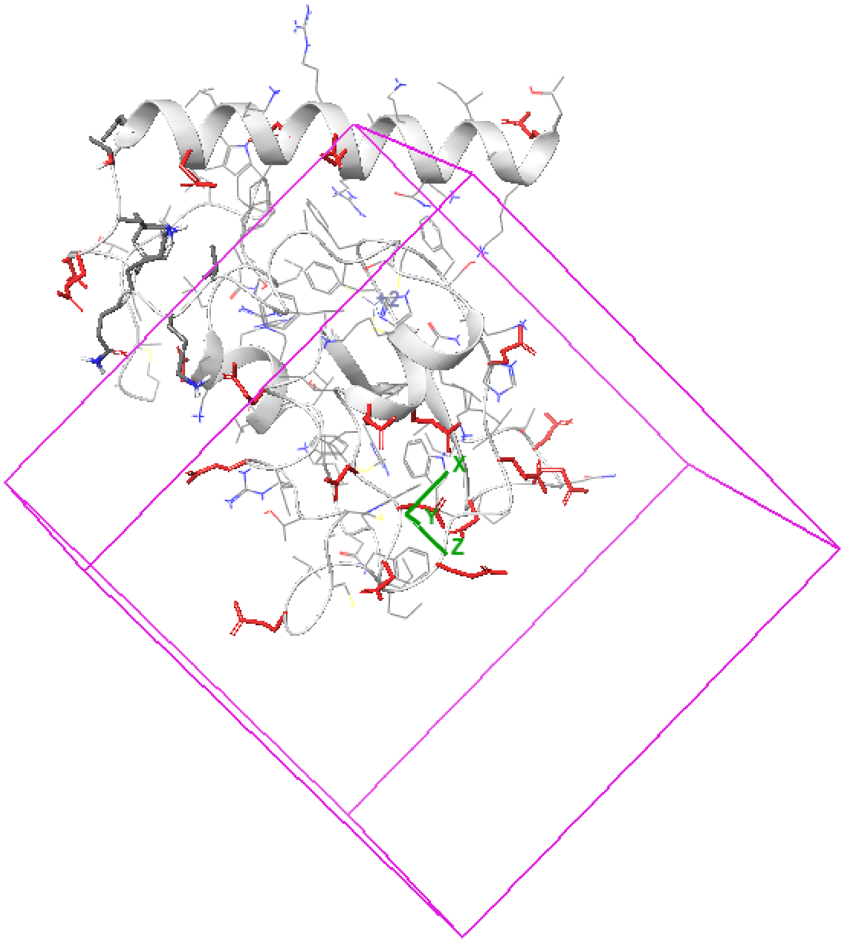


The prepared ligands and the generated grid were used for docking (Ligand Docking). The method was extra precise and the ligand sampling flexible. The following conditions were chosen: sample nitrogen inversions and ring conformation, bias sampling of torsions for amides and add Epik state penalties to docking score.

**Table S1:** Docking scores of GCP ligands on Survivin's Histone H3 binding site.

| Ligand | Docking score | |
|---|---|---|
| gcpAalagcp | -5.451 | =Ligand **L1** |
| gcpAalagcpBz | -5.445 | |
| gcpAalagcpBz | -5.350 | |
| gcpAalagcp | -5.275 | |
| gcparg | -5.255 | |
| gcpAalagcptBu | -5.193 | |
| gcplys | -5.138 | |
| gcplys | -5.021 | |
| gcpAalagcptBu | -4.931 | |
| lyslys | -4.786 | |
| lysarg | -4.676 | |
| gcpglu | -4.444 | |
| gcpAala | -4.405 | |
| argarg | -4.369 | |
| lysarg | -4.351 | |
| lysarg | -4.266 | |
| lysarg | -4.184 | |
| lyslys | -4.158 | |
| argarg | -4.155 | |
| lysarg | -4.144 | |
| gcparg | -4.132 | |
| gcpgln | -4.128 | |
| lysarg | -4.095 | |
| argarg | -4.007 | |
| argarg | -3.901 | |
| gcpAala | -3.895 | |
| lysarg | -3.885 | |
| argarg | -3.820 | |
| lyslys | -3.747 | |
| gcpgln | -3.716 | |
| lysgcp | -3.693 | |
| argarg | -3.683 | |
| lysgcp | -3.658 | |
| argarg | -3.635 | |
| lyslys | -3.632 | |
| lyslys | -3.568 | |
| gcpthr | -3.558 | |
| argarg | -3.524 | |
| gcpthr | -3.514 | |
| gcpglu | -3.496 | |
| lysgcp | -3.395 | |
| lysgcp | -3.234 | |
| gcpphe | -3.186 | |
| gcpAala | -3.072 | |
| lysarg | -2.913 | |
| lyslys | -2.788 | |
| gcpAala | -2.688 | |
| lysgcp | -2.679 | |
| lyslys | -2.599 | |
| lyslys | -2.541 | |
| gcpphe | -2.534 | |
| gcpAala | -2.491 | |
| gcpgly | -2.374 | |
| lysgcp | -2.337 | |
| lysgcp | -2.293 | |
| gcpAala | -2.120 | |
| gcpAala | -1.932 | |
| lysgcp | -1.852 | |
| gcpAala | -1.348 | |

## 14. References

[1] C. Schmuck, Carboxylate Binding by 2-(Guanidiniocarbonyl) Pyrrole Receptors in Aqueous Solvents: Improving the Binding Properties of Guanidinium Cations through Additional Hydrogen Bonds, Chem. Eur. J., **2000**, *6*, 709-718.

[2] A. Favier, B. Brutscher, Recovering lost magnetization: polarization enhancement in biomolecular NMR, *J. Biomol. NMR,* **2011,** *49*, 9.

[3] R .L. J. Keller, Optimizing the process of NMR spectrum analysis and computer aided resonance assignment (PhD thesis), **2004**, ETH Zürich, Zürich, Switzerland.

[4] C. Sun, D. Nettesheim, Z. Liu, E. T. Olejniczak, Solution Structure of Human Survivin and Its Binding Interface with Smac/Diablo, Biochemistry, **2005**, *44*, 11.

[5] A. Ayed, F. A. Mulder, G. S. Yi, Y. Lu, L. E. Kay, C. H. Arrowsmith, *Nat. Struct. Biol.,* **2001**, *8*, 756.
